# Exploratory Metabolomics Profiling in the Kainic Acid Rat Model Reveals Depletion of 25-Hydroxyvitamin D3 during Epileptogenesis

**DOI:** 10.1038/srep31424

**Published:** 2016-08-16

**Authors:** Svenja Heischmann, Kevin Quinn, Charmion Cruickshank-Quinn, Li-Ping Liang, Rick Reisdorph, Nichole Reisdorph, Manisha Patel

**Affiliations:** 1 Department of Pharmaceutical Sciences, University of Colorado, School of Pharmacy, 12850 East Montview Boulevard, Aurora, CO 80045, USA; 2 Department of Immunology, National Jewish Health, 1400 Jackson Street, Denver, CO 80206, USA

## Abstract

Currently, no reliable markers are available to evaluate the epileptogenic potential of a brain injury. The electroencephalogram is the standard method of diagnosis of epilepsy; however, it is not used to predict the risk of developing epilepsy. Biomarkers that indicate an individual’s risk to develop epilepsy, especially those measurable in the periphery are urgently needed. Temporal lobe epilepsy (TLE), the most common form of acquired epilepsy, is characterized by spontaneous recurrent seizures following brain injury and a seizure-free “latent” period. Elucidation of mechanisms at play during epilepsy development (epileptogenesis) in animal models of TLE could enable the identification of predictive biomarkers. Our pilot study using liquid chromatography-mass spectrometry metabolomics analysis revealed changes (p-value ≤ 0.05, ≥1.5-fold change) in lipid, purine, and sterol metabolism in rat plasma and hippocampus during epileptogenesis and chronic epilepsy in the kainic acid model of TLE. Notably, disease development was associated with dysregulation of vitamin D3 metabolism at all stages and plasma 25-hydroxyvitamin D3 depletion in the acute and latent phase of injury-induced epileptogenesis. These data suggest that plasma VD3 metabolites reflect the severity of an epileptogenic insult and that a panel of plasma VD3 metabolites may be able to serve as a marker of epileptogenesis.

Epilepsy can be acquired or inherited^1^. Acquired epilepsies account for about 60% of all cases. The most common form of acquired epilepsy, temporal lobe epilepsy (TLE), often occurs after brain injury. It is characterized by spontaneous recurrent seizures that arise after a variable latent period following the inciting insult^2^. Known risk factors include infection, trauma, stroke, and fever; however, predicting epileptogenic potential in the subset of at-risk patients is difficult. To date, the electroencephalogram (EEG) is the only objective clinical tool for diagnosing epilepsy^3^,^4^. Blood can provide more comprehensive information and sampling is minimally invasive, therefore, blood is a very desirable medium for clinical diagnostics. Measurement of blood cholesterol and hemoglobin A1c serves to assess the risk of coronary artery disease^5^ and parameters of glucose metabolism in diabetes patients^6^, respectively. Similarly, several blood-derived protein and metabolite biomarkers have been proposed in neurodegenerative diseases^7^,^8^, including 25-hydroxy vitamin D3 (25-(OH)-VD3)^9^, the clinically measured precursor of the active form of vitamin D3 (VD3), 1,25-dihydroxyvitamin D3 (1,25-(OH)_2_-VD3). Despite considerable knowledge about metabolic, mitochondrial, and redox changes^2^,^10^,^11^ that arise in the brain following epileptogenic injury, it is unknown how an injury induces chronic epilepsy. Pathway alterations have been described, but are poorly understood^12^,^13^. Metabolomics approaches in models of TLE could uncover metabolic signatures, i.e. biomarkers that represent global biochemical changes in TLE and predict disease state and response to treatment. Biomarkers and knowledge of pathway alterations could offer possibilities for early and specific diagnosis of TLE; this could aid the development of disease-modifying therapies and eventually help to prevent the development of chronic epilepsy after an inciting insult.

The collection of small molecules present in an organism or in one of its distinct matrices, i.e. body fluid, cell lysate, or tissue, is referred to as its metabolome^14^. The metabolome captures the state of the system and can enable one to differentiate between conditions of health and disease by discriminating between distinct signatures. Mass spectrometry (MS)-based metabolomics studies can offer insights into the etiology of TLE, suggest biomarkers that potentially predict its onset and/or severity, and can lead the way to early intervention before epilepsy manifests. It is likely that no single molecule, but rather a panel of metabolites is required to predict disease onset and/or progression. Using a non-targeted MS-based approach, we profiled plasma and hippocampal tissue from the kainic acid (KA) rat model of TLE. The ultimate goal of this study was to identify signatures that potentially predict disease onset, severity, and/or suggest metabolic routes than could be exploited for intervention.

## Results

### Study design

The goal of this study was to determine metabolic changes occurring in hippocampus and plasma of rats following chemoconvulsant-induced epileptogenic injury. Three time points of analysis were selected based on previous studies^15^ to reflect different phases of disease development/manifestation following an epileptogenic insult (SE): acutely after SE (48 h or acute), latent phase devoid of behavioral seizures (1 wk or latent), and chronically epileptic, the phase in which spontaneous seizures occur (12 wk or chronic).

### Overall metabolic changes in hippocampus and plasma

Compounds that were altered with a p value < 0.05 and exhibited >1.5-fold change between groups are referred to as “changing” compounds/“changed” in the following unless otherwise stated.

**Figure 1:**
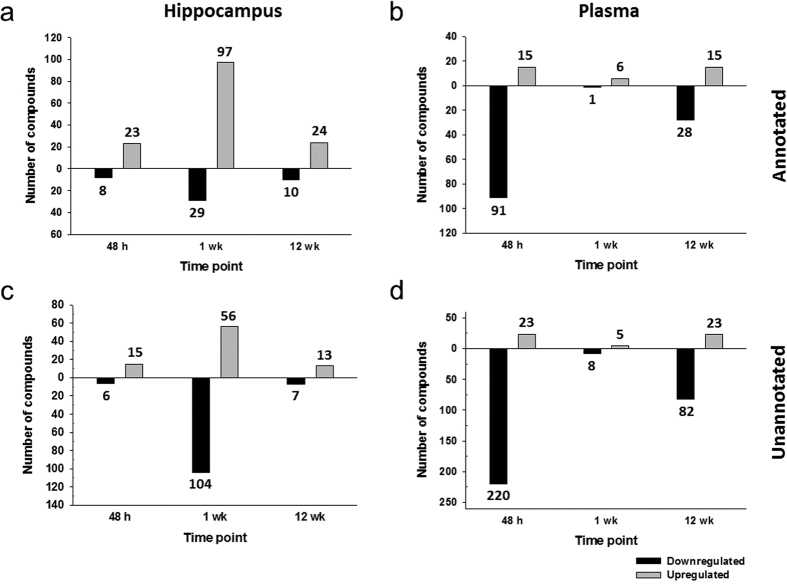
Changes (p < 0.05, >1.5-fold change) in metabolites in rats treated according to the KA model of TLE at acute, latent, and chronic time points. (**a**) Changes in annotated compounds in the hippocampus. (**b**) Changes in annotated compounds in plasma. (**c**) Changes in unannotated compounds in the hippocampus. (**d**) Changes in unannotated compounds in plasma. Statistical analysis by Student’s unpaired t-tests and Benjamini-Hochberg false discovery rate multiple testing correction using asymptotic p-value computation.

1178 metabolites were found in hippocampal samples (lipid fraction: 1002, aqueous fraction: 176) and 1432 in plasma samples (lipid fraction: 1110, aqueous fraction: 321). A total of 268 annotated (compound annotations are based on isotope ratios, accurate mass, chemical formulae, and scores as well as the use of specific databases as described in the Methods section) and 464 unannotated (compounds that could not be annotated by database searches and are represented by a formula or compound number and retention time as described in the Methods section) metabolites were changed at the three time points in both matrices; these are listed in Supplementary Tables S1 (annotated) and S2 (unannotated). Figure 1 shows the number of changing (up- or downregulated) compounds in the two matrices at the three time points.

Changes were measured predominantly in plasma at acute and chronic time points, whereas most changes at the latent time point were measured in the hippocampus. While downregulation of metabolites was universal at the acute and chronic time points in plasma, an increase of differentially regulated metabolites was observed at 1 wk (latent period) in the hippocampi of KA animals vs. controls.

Venn diagrams reflecting the overlap and uniqueness of changing compounds in different matrices at the three time points are shown in Supplementary Fig. S1. Lists of compounds (unique and overlap) for each Venn diagram can be found in Supplementary Tables S3 (annotated compounds) and S4 (unannotated compounds); an analysis of overlap between all six groups (hippocampus vs. plasma, 48 h vs. 1 wk. vs. 12 wk) is listed in Supplementary Table S5 (annotated compounds). Only 12 compounds that were found to change in the hippocampus also changed in plasma, i.e. hypoxanthine, Cer(d18:0/24:1(15Z)), citrulline, PC(18:2(2E,4E)/0:0), PC(20:0/0:0), 11Z-octadecenylcarnitine, proline, tetrahydrodeoxycorticosterone, docosahexaenoic acid (DHA), palmitoylcarnitine, GlcCer(d18:1/24:0), and PC(O-18:1(1E)/0:0).

More changing compounds overlapped between time points in the hippocampus (Supplementary Fig. 1, Panels a and d) than in plasma (Supplementary Fig. 1, Panels b and e). Patterns of overlap of metabolites are variable between both matrices. Considerable overlap, i.e. shared changes, between 48 h (acute) and 1 wk (latent) time points as well as 1 wk (latent) and 12 wk (chronic) time points in the hippocampus, was found, whereas minimal overlap of changes in plasma was seen. On the other hand, 48 h and 12 wk time points showed a bigger overlap in plasma than in the hippocampus. Only a small number of annotated metabolites was found to change in both matrices; no unannotated metabolites were changed in both matrices. One reason for the missing overlap of unannotated compounds between matrices may be that several compounds are depicted by compound number and retention time instead of an assigned formula. Compound numbers are unique to the respective data analysis workflow and automatically assigned by MPP (i.e. unique for hippocampus lipid or aqueous, plasma lipid or aqueous) and do not match-up across analyses. In addition, retention times for liquid chromatography (LC) separation for tissue and plasma differed slightly, although identical gradients were used. Compounds depicted by compound number and retention time, therefore, cannot be matched across matrices and are counted as unique elements in Supplementary Fig. 1 and Supplementary Table S4.

The number of metabolic differences also varied between time points, depending on the matrix. Thirteen metabolites were common between the 48 h (acute) and 1 wk (latent) time point, 13 metabolites were common to both 1 wk (latent) and 12 wk (chronic) time points, and no metabolites were common between 48 h (acute) and 12 wk (chronic) time points in the hippocampus. Conversely, only one common metabolite was found between 48 h (acute) and 1 wk (latent), only one common metabolite between 1 wk (latent) and 12 wk (chronic) time points, but 11 common metabolites between 48 h (acute) and 12 wk (chronic) time points in plasma (Supplementary Fig. 1, for a detailed list of metabolites and their overlap between time points please see Supplementary Table S3). Fourteen changing metabolites common to all examined time points were found in hippocampus vs. none that were common to all time points in plasma.

An overview of regulation showing inconsistency and persistency of changes of metabolites across time points is given in Supplementary Table S5. Only the seven following metabolites showed oppositional changes in KA animals vs. controls across time points: hypoxanthine (Hip 48 down vs. Hip 1 wk/12 wk up and Plas 48 h down vs. Plas 12 wk up), 1,4-beta-D-glucan (Hip 48 h down vs. Hip 1 wk up), Tyr-Thr-OH (Hip 48 h down vs. Hip 1 wk/12 wk up), PC(18:2(2E,4E)/0:0), PC(20:0/0:0), L-proline (Hip 1 wk up vs. Plas 48 h down), and PC(O-18:1(1E)/0:0) (Hip 1 wk up vs. Plas 12 wk down). All other changes in annotated, differentially regulated metabolites that occurred across time points or matrices were persistent in their regulation.

### Changes in specific metabolites and metabolite groups

#### Overall changes

**Figure 2:**
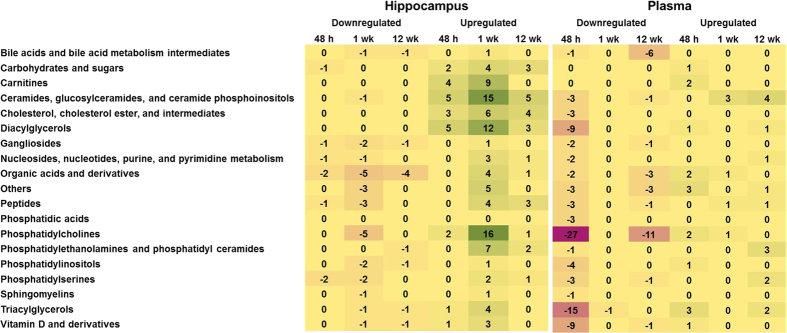
Heat maps of changing hippocampal and plasma metabolites sorted according to designated categories at three examined time points. Statistical analysis by Student’s unpaired t-tests and Benjamini-Hochberg false discovery rate multiple testing correction using asymptotic p-value computation.

Figure 2 shows heat maps for numbers of metabolites changed in hippocampi and plasma of rats at the three examined time points grouped according to their designated categories. In the hippocampus, the most prominent changes occurred in the groups “Ceramides, glucosylceramides, and ceramide phosphoinositols”, “Diacylglycerols”, and “Phosphatidylcholines”, which were mainly upregulated at 1 wk. Major changes in plasma occurred in “Phosphatidylcholines”, “Triacylglycerols”, “Diacylglycerols”, and “Vitamin D and derivatives” after 48 h, and in “Phosphatidylcholines” and “Bile acids and bile acid metabolism intermediates” after 12 wk.

#### Changes in VD3 metabolites and 25-(OH)-VD3

**Figure 3:**
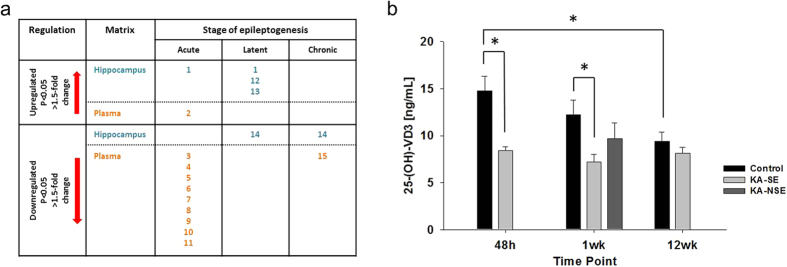
Changes in vitamin D metabolites and 25-(OH)-VD3. (**a**) Changes in hippocampal tissue (blue) and plasma (orange) in vitamin D metabolites and precursors of rats 48 h, 1 wk, and 12 wk post KA treatment. Numbers represent specific metabolites: (1) 1alpha-hydroxy-26,27-dinorvitamin D3 25-carboxylic acid, (2) 1alpha,25-dihydroxy-19-nor-22-oxavitamin D3, (3) 1-Hydroxyvitamin D3 diacetate, (4) 2alpha-(3-Hydroxypropyl)-1alpha,25-dihydroxy-19-norvitamin D3, (5) 1,25-Dihydroxyvitamin D3 3-glycoside, (6) 1alpha,25-dihydroxy-24a,24b-dihomovitamin D3, (7) 1α,25-dihydroxy-26,27-dimethylvitamin D3, (8) 1α-hydroxy-26,27-dimethylvitamin D3, (9) 2α-(3-Hydroxypropyl)-1α,25-dihydroxy-19-norvitamin D3, (10) 2beta-methyl-3-epi-1beta,25-dihydroxyvitamin D3, (11) 1alpha,25-dihydroxy-26-methylvitamin D3, (12) 3-Deoxyvitamin D3, (13) 1α,25-dihydroxy-24a,24b,24c-trihomo-22-thia-20-epivitamin D3, (14) 7-dehydrocholesterol (Provitamin D3), (15) 26,27-diethyl-1α,25-dihydroxy-22-thia-20-epivitamin D3. Compounds were tentatively identified using exact mass and isotope ratios. Alternative vitamin D metabolites are listed in Supplementary Table S6. (**b**) 25-(OH)-VD3 concentrations in plasma of KA-treated rats that experienced SE (KA-SE), no-SE (NSE), and controls measured by LC-MS/MS. Statistical analysis by Student’s unpaired t-tests and Benjamini-Hochberg false discovery rate multiple testing correction using asymptotic p-value computation for the comparison of two groups (**a**) and by one-way ANOVA combined with Tukey’s *post hoc* test for the comparison of more than two groups (**b**). 25-(OH)-VD3 levels of NSE sacrificed at the 1 wk time point were not significantly different to either controls or KA-SE.

It should be noted that many publications do not distinguish between VD2 and VD3 and use the general term “vitamin D” (VD). Our results and discussion generally relate to VD3 unless otherwise noted. In case the term VD is used, no distinction was made between VD2 and VD3. Significant changes in several VD3 metabolites and 7-dehydrocholesterol, a precursor of 25-(OH)-VD3 and VD3 metabolites, were detected in hippocampi and/or plasma of KA-treated rats at all assessed time points. Changes related to VD3 metabolites and 7-dehydrocholesterol are summarized in Fig. 3, Panel a. Annotations are based on exact mass and isotopic distribution (Metabolomics Standard Initiative level 2^16^), therefore multiple compounds are possible for a respective sum formula. The listed VD3 metabolites are only examples for possible structures. Due to the metabolic role VD3 plays, the potential role of its metabolites, and their biomarker potential, Supplementary Table S6 lists all other possible annotations (limited to VD3 metabolites in general and to VD3 metabolites and cholesterol and derivatives for 7-dehydrocholesterol in particular).

#### Validation of results related to changes in VD3 metabolism by measurement of 25-(OH)-VD3

Since an upregulated VD3 brain metabolism with concomitant depletion of VD3 plasma metabolites was apparent (Fig. 3, Panel a), measurement of 25-(OH)-VD3, the routinely measured precursor of 1,25-(OH)_2_-VD3 in the clinic, by a quantitative high performance liquid chromatography (HPLC)-MS/MS assay was used to further validate our findings. This analysis revealed significant downregulation of 25-(OH)-VD3 at the acute (p = 0.012; controls: 14.80 ± 1.50 ng/mL, KA: 8.44 ± 0.39 ng/mL) and latent (p = 0.026; controls: 12.25 ± 1.52 ng/mL, KA: 7.19 ± 0.82 ng/mL) time point in the KA group (Fig. 3, Panel b). Non-SE (NSE) animals, which were sacrificed at the 1 wk time point, showed 25-(OH)-VD3 levels of 9.67 ± 1.70 ng/mL with no significant difference compared to controls (p = 0.30) or the KA-SE group (p = 0.24). To determine if hemodynamic and circulatory effects of SE per se decreased plasma 25-(OH)-VD3, we measured plasma 25-(OH)-VD3 after 6 h of SE in a separate cohort of rats (controls n = 4, KA-treated n = 8). No significant changes (p = 0.81) occurred comparing 25-(OH)-VD3 plasma levels of controls (9.05 ± 0.29 ng/mL) with KA-treated (8.44 ± 0.6 ng/mL) animals. Interestingly, a significant decrease in plasma 25-(OH)-VD3 occurred in the control groups over time i.e. between the 48 h (acute) and 12 wk (chronic) animals (p = 0.021, 48 h: 14.80 ± 1.50 ng/mL, 12 wk: 9.40 ± 1.98 ng/mL), such that no changes in plasma 25-(OH)-VD3 were observed between treatment groups (controls vs. KA) at the chronic time point (p = 0.333; controls: 9.40 ± 1.98 ng/mL, KA: 8.15 ± 1.55 ng/mL). One explanation for these observations may be a tight homeostatic control of VD3 metabolism such that a maximal depletion produced by either injury or aging was reached. To determine if this was the case, we asked whether maintaining rats in our vivarium for the same time span as our chronic cohort, i.e. 14 wk total would result in depletion of 25-(OH)-VD3 levels as acute treatment of KA. Indeed, we observed that a 14 wk period in the vivarium resulted in significant depletion of plasma 25-(OH)-VD3 levels in control rats (9.05 ± 0.29 ng/mL vs. 6.10 ± 0.35 ng/mL, p < 0.001). Furthermore, treatment with KA of this group of rats did not result in any further depletion of plasma 25-(OH)-VD3 levels 48 h later (14 wk controls: 6.10 ± 0.35 ng/mL, n = 3; KA-treated: 5.84 ± 0.40 ng/mL, n = 5; p = 0.68).

### Pathway analysis

**Figure 4:**
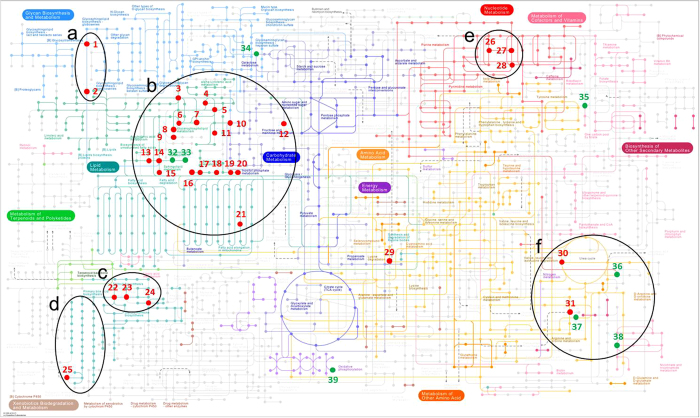
KEGG map “Metabolic pathways”. Metabolites that were significantly changed (p < 0.05) in hippocampus and/or plasma at the 48 h, 1 wk, and/or 12 wk time point are marked and color-coded according to fold-change (red: >1.5-fold change, green: <1.5-fold change). Metabolic sites of major dysregulation of metabolites are circled. Numbers code for the following KEGG identifiers/compounds: 1: C01290 (Lactosylceramide), 2: C01190 (Glucosylceramide), 3: C00157 (Phosphatidylcholine), 4: C01885 (1-Acylglycerol), 5: C00416 (Phosphatidate), 6: C00350 (Phosphatidylethanolamine), 7: C02737 (Phosphatidylserine), 8: C00422 (Triacylglycerol), 9: C04046 (Monoglucosyldiacylglycerol), 10: C01194 (Phosphatidylinositol), 11: C03892 (Phosphatidylglycerophosphate), 12: C00352 (D-Glucosamine phosphate), 13: C12126 (Dihydroceramide), 14: C00195 (Ceramide), 15: C00550 (Sphingomyelin), 16: C04635 (1-Alkenylglycerophosphoethanolamine), 17: C04756 (Phosphatidalethanolamine), 18: C00958 (1-Alkenyl-2-acylglycerophosphocholine), 19: C04317 (1-Alkyl-2-lyso-sn-glycero-3-phosphocholine), 20: C05212 (2-Acyl-1-alkyl-sn-glycero-3-phosphocholine), 21: C05273 ((2E)-Tetradecenoyl-CoA), 22: C00187 (Cholesterol), 23: C01164 (7-Dehydrocholesterol), 24: C01561 (25-Hydroxyvitamin D3), 25: C00695 (Cholic acid), 26: C00212 (Adenosine), 27: C00294 (Inosine), 28: C00262 (Hypoxanthine), 29: C00956 (L-2-Aminoadipic acid), 30: C00327 (L-Citrulline), 31: C00148 (L-Proline), 32: C02686 (Galactosylceramide), 33: C06125 (Galactosylceramidesulfate), 34: C00124 (D-Galactose), 35: C00255 (Riboflavin), 36: C00086 (Urea), 37: C00170 (5′-Methylthioadenosine), 38: C00153 (Nicotinamide), 39: C00009 (Phosphoric acid). Letters code for metabolic sites of dysregulation: (**a**) Glycosphingolipid biosynthesis, (**b**) Lipid metabolism (glycerophospholipid metabolism, glycerolipid metabolism, sphingolipid metabolism, ether lipid metabolism), (**c**) Vitamin D metabolism, (**d**) Bile acid metabolism, (**e**) Nucleotide metabolism, (**f**) Amino acid metabolism and urea cycle. Statistical analysis by Student’s unpaired t-tests and Benjamini-Hochberg false discovery rate multiple testing correction using asymptotic p-value computation.

Figure 4 shows the KEGG reference pathway “Metabolic Pathways” with metabolites marked that were significantly changed at p < 0.05 and >1.5-fold (red; Supplementary Table S1) or <1.5-fold (green, Supplementary Table S7; added for pathway coverage and completeness) at the 48 h, 1 wk, and/or 12 wk time point. Areas of major dysregulation of metabolites are circled. For a more detailed overview of changes associated with matrix and time point, please refer to the heat maps in Fig. 2.

Pathway analysis by MetaboAnalyst was conducted for each time point based on combined changes within both matrices, i.e. changes in both hippocampus and plasma combined, at each time point individually. Pathway analysis was also conducted on all time points and matrices combined for better coverage and simplified presentation of data (Supplementary Table S8). All pathways matched by MetaboAnalyst with ≥3 hits are depicted in Supplementary Figs S2–8. Supplementary Tables S1 and S7 list significantly changed metabolites with >1.5-fold chance and <1.5-fold change, respectively. Heat maps indicate numbers of changed metabolites, direction, time point, and matrix of change.

#### Glycerophospholipid, sphingolipid, ether lipid, and glycerolipid metabolism

The most robust changes in the hippocampus occurred at 1 wk in glycerophospholipid, sphingolipid, ether lipid, and glycerolipid metabolism (Supplementary Figs S2–S5) with downregulation of phosphatidylcholines (4 downregulated; Supplementary Fig. S2), upregulation of acylglycerophosphhocholines (3 upregulated; Supplementary Fig. S2), upregulation of ceramides (9 upregulated, Supplementary Fig. S3), glucosyl- (3 upregulated) and lactosylceramides (2 upregulated; Supplementary Fig. S3), upregulation of acyl-alkyl-sn-glycerophosphatidylcholines (5 upregulated; Supplemenary Fig. S4), and triacylglycerols (4 upregulated; Supplementary Fig. S5). Robust changes in plasma occurred at 48 h and 12 wk with downregulation of acylglycerophosphocholines (18+2 downregulated at 48 h and 7 downregulated at 12 wk; Supplementary Fig. S2), upregulation of glucosylceramides (3 upregulated at 1 wk; Supplementary Fig. S3) and ceramides (3 upregulated at 12 wk; Supplementary Fig. S3), downregulation of acyl-alkyl-sn-glycerophosphatidylcholines (4 downregulated; Supplementary Fig. S4) at 48 h, and downregulation of triacylglycerols (15 downregulated, Supplementary Fig. S5) at 48 h.

#### Purine and amino acid metabolism

The largest changes in purine metabolism occurred at 1 wk in the hippocampus with downregulation of adenosine and concomitant upregulation of the downstream metabolites inosine and hypoxanthine (Supplementary Fig. S6). Changes in hypoxanthine levels in the hippocampus were also measured at 48 h (downregulation) and 12 wk (upregulation), these changes were seen in plasma as well. Proline was downregulated in plasma at 48 h, while increased in the hippocampus at 1 wk (Supplementary Fig. S7). Citrulline was downregulated in hippocampus and plasma at 48 h. 4-aminobutyraldehyde was decreased in the hippocampus at 1 wk.

#### Steroid metabolism

**Figure 5:**
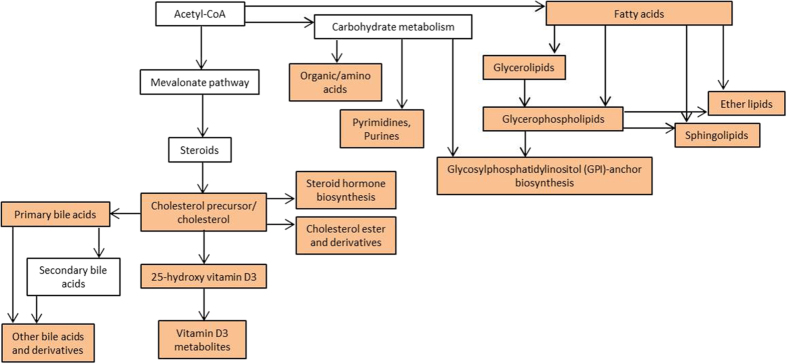
Interrelation of pathways and metabolite groups. Pathways and metabolite groups that showed changes in metabolite concentrations at at least one of the examined time points are marked in red. Pathway was adapted from KEGG. For details on metabolites, regulation, time point or matrix of change please see Fig. 2 and Supplementary Figs S2–S8.

Changes in the biosynthesis of cholesterol and downstream metabolites (Figs 2 and 5, Supplementary Fig. S8) such as cholesterol ester, bile acids (Fig. 2 and Supplementary Table S1), 7-dehydrocholesterol, and VD3 metabolites (Fig. 3 and Supplementary Table S6) are prevalent at all stages of epileptogenesis in both matrices. Cholesterol was upregulated in the hippocampus at 1 wk post-treatment, cholesterol ester were increased at 48 h (3 upregulated) and 1 wk (5 upregulated) but decreased at 12 wk (4 downregulated; Supplementary Fig. S8). Bile acids were markedly downregulated in the chronic phase in plasma. VD3 metabolites were downregulated in the acute phase in plasma. Tetrahydrodeoxycorticosterone (THDOC) was upregulated in plasma at 48 h and in the hippocampus at 1 wk while 12alpha-methylpregna-4,9(11)-diene-3,20-dione was downregulated in the hippocampus at 1 wk.

### Relationship of pathways, metabolite groups, and specific metabolites

Figure 5 shows the downstream relationship of pathways and metabolite groups that were affected in the studied time course. Overall, the major pathways changing in this model of TLE with strong relation to epileptogenesis are pathways of lipid metabolism, purine metabolism, cholesterol/steroid biosynthesis, and subsequent VD3 metabolism.

## Discussion

Considerable changes occurred in brain and plasma metabolites in the KA model of TLE throughout the stages of epilepsy development. It should be pointed out that epileptogenesis may be a continuous and progressive process in chemoconvulsant models with a variable latent period^17^. In our laboratory the 1 wk time point after KA injection is best associated with low seizure probability by EEG and behavior associated with a latent period.

The distinct patterns of changes within the two matrices might reflect different aspects of disease development and manifestation. The overlap of metabolites that occurs between the acute and latent as well as latent and chronic phases (but not between acute and chronic phases) in the hippocampus could indicate that disease progresses continuously in the hippocampus. Accordingly, certain changes in the hippocampus span adjacent phases in the KA model of TLE. On the other hand, plasma shows a pronounced overlap between the two time points where seizures are present (acute and chronic), which could reflect systemic effects of seizure activity or seizure-dependent changes. Many changes arising in the hippocampus vs. minimal changes in plasma in the latent phase of epileptogenesis are likely due to the fact that the brain is the focus of disease development and progression. The minimal overlap of changes in the hippocampus and plasma (12 compounds) could be due to the fact that epilepsy is a brain-specific disorder and that many compounds do not cross the blood-brain barrier. Decreases in plasma metabolites at the acute time point could be due to the animals’ response to treatment independent of epilepsy development, i.e. SE and decreased food and water intake. Especially, decreases in diacylglycerols (DAGs), triacylglycerols (TAGs), and peptides could reflect diminished food intake typically observed following SE. If energy metabolism is compromised due to fasting or starvation the brain becomes reliant on fatty acids as energy substrates^18^. Elevated acylcarnitine levels at 48 h in the hippocampus could reflect an increased demand of fatty acids as alternative energy source.

Although striking changes occurred within certain metabolite groups, no statistically significant differences were found during pathway analysis for these specific groups. This could be due to the incompleteness of pathway information, the lack of pathway data, and inadequate metabolome coverage; a problem in current metabolomics research that is discussed elsewhere^19^. Changes in several classes of metabolites are notable; DAGs were upregulated at all time points in the hippocampus, but decreased in plasma after 48 h (9 downregulated; Fig. 2), phosphatidic acids (PAs) were decreased in plasma after 48 h (3 downregulated, Fig. 2). DAGs and PAs are implicated in calcium release and subsequent cellular responses such as neurotransmitter release via vesicular exocytosis in neurons^20^,^21^. Acylcarnitines (upregulated in acute and latent phase in the hippocampus and acute phase in plasma, Fig. 2) are necessary to maintain mitochondrial function and for the transport of long-chain fatty acids from the cytosol into mitochondria or peroxisomes and consecutive fatty acid beta-oxidation^22^. It has been proposed to assess overall changes in beta-oxidation and energy consumption through monitoring of plasma carnitines and acylcarnitines, in particular, plasma acetylcarnitine (upregulated at 1 wk in the hippocampus) and palmitoylcarnitine (upregulated at 48 h in plasma and 1 wk in the hippocampus)^22^. Upon decrease in fatty acid beta-oxidation an increase in long-chain acylcarnitines (e.g. palmitoylcarnitine) would be expected at the acute time point. Upregulated acylcarnitines in acute and latent phases could reflect an increased demand of acetyl-CoA from beta-oxidation to meet the brain’s energy requirements. Besides their role in beta-oxidation carnitines have been implicated in the development of neurological diseases and neuroprotection^18^.

Other changing compounds with potential relation to epileptogenesis are aminoadipic acid (downregulated at 1 wk in the hippocampus) and DHA (upregulated in plasma at 48 h and in the hippocampus at 1 wk). Aminoadipic acid has neuroexitatory properties as it is a regulator of kynurenic acid production in the hippocampus. Kynurenic acid is an excitatory amino acid receptor antagonist and potentially has protective effects^23^. DHA is the most abundant polyunsaturated acid in the brain and is involved in brain development^24^. DHA has recently been suggested as an interventional treatment of seizures in humans as it exerts antiepileptic effects in rodent models. Upregulation could show an increased demand for this compound at early stages of epileptogenesis.

Our data show downregulation of nine plasma VD3 metabolites in the acute phase (Fig. 3, Panel a), one metabolite is upregulated in both plasma and in the hippocampus (Fig. 3, Panel a). In the latent phase, 7-dehydrocholesterol is downregulated in the hippocampus; 7-dehydrocholesterol is the direct precursor of VD3 and an indirect precursor of 1,25-(OH)_2_-VD3, which is the active form of VD3. Three VD3 metabolites are upregulated in the latent phase in the hippocampus. Similarly, the chronic time point shows downregulation of 7-dehydrocholesterol and concomitant downregulation of one VD3 metabolite in plasma. Our data show, for the first time, dysregulation of several VD3 metabolites at the acute, latent, and chronic phases of injury-induced epileptogenesis/epilepsy in plasma and hippocampus. We also show for the first time concomitant downregulation of 25-(OH)-VD3 at acute and latent stages in plasma. Our data suggests that the decrease in plasma 25-(OH)-VD3 is caused by epileptogenic injury; possibly due to an increased demand of VD3. This is supported by the finding that significant depletion of 25-(OH)-VD3 was not observed in NSEs (KA-treated but no SE) which do not develop full SE or chronic epilepsy. Turnover of brain VD3 may be altered in a compensatory manner as affirmed by upregulation of hippocampal VD3 metabolites (acute and latent) and downregulation of hippocampal 7-dehydrocholesterol (latent and chronic).

Plasma VD depletion is common in epileptic patients, but has traditionally been attributed to anti-epileptic drug regimens^25^ rather than disease pathogenesis. However, the VD endocrine system has been suggested to be involved in the regulation of seizures and to potentially represent a drug target to treat epileptic disorders, as VD has neuroprotective and immunomodulatory properties^26^. 1,25-(OH)_2_-VD3 has been shown to decrease both the expression of inducible nitric oxide synthase (iNOS)^27^,^28^ and oxidative stress^29^,^30^; the latter is a factor that significantly contributes to mitochondrial dysfunction and epileptogenesis^2^,^11^. In addition, VD is thought to have other neuroprotective and immunomodulatory properties^26^. It also is involved in brain development, e.g. the regulation of brain cell proliferation and differentiation. Several studies show evidence for strong links between VD/VD metabolite levels and the course of epileptogenesis, seizure severity, and seizure frequency. Christiansen *et al*. reported that VD2 administration reduced seizure frequency in epilepsy patients by 30%^31^. Siegel *et al*. demonstrated that 1,25-(OH)_2_-VD3 delivered directly to the hippocampus increases the threshold for seizures triggered by electrical stimulation in rats^32^. Hippocampal injection of 1,25-(OH)_2_-VD3 prior to seizure induction showed anticonvulsant effects in mice^33^ and an increased severity of chemically induced seizures was seen in VD receptor knockout mice^34^. In a more recent study, Hollo *et al*. showed that normalization of serum 25-(OH)-VD3 levels causes a reduction in seizure frequency of 40% in patients with pharmacoresistant epilepsy^35^. Further evidence of VD’s protective effects is described elsewhere in the literature^33^,^36^,^37^. As additional (demographic) evidence that VD is a major mediator of seizure manifestation, seasonal patterns of seizure onset and frequency have been reported, as well as a correlation of seizures and latitude^38^.

Currently there are no molecular markers available to evaluate the epileptogenic potential of an insult^13^. Biomarkers that can quickly and reliably assess the risk of individuals to develop epilepsy, especially peripheral/blood biomarkers, are essential. Biomarker discovery poses difficulties as epilepsy is a brain-specific disorder and many compounds do not cross the blood-brain barrier^39^. Our discovery that 25-(OH)-VD3 decreases in the acute and latent phases of SE-induced epileptogenesis and related metabolites are dysregulated in phases of epileptogenesis and chronic epilepsy suggests that 25-(OH)-VD3 and related metabolites may be candidates for a biomarker panel. Dysregulation of 25-(OH)-VD3 and VD3 metabolism is linked to a variety of conditions^9^. Before 25-(OH)-VD3 and/or VD3 metabolites can be further validated as markers or as parts of marker panels to assess the epileptogenic potential of an injury in animal models and potentially consecutive clinical trials, several issues need careful consideration. First, since VD3 deficiency is common and many factors influence VD3 levels, e.g. gender, age, ethnicity, season, a panel of metabolites may be required to serve as a marker of the epileptogenic potential of a brain injury rather than an individual metabolite. This panel of metabolites could include 25-(OH)-VD3 in addition to 7-dehydrocholesterol and other VD3 plasma metabolites (or their alternatives, Supplementary Table S6) observed to change in this study (Supplementary Table S1). Secondly, dysregulation of downstream VD3 metabolites or a ratio of precursor to (a) downstream metabolite(s) may reflect more accurately the turnover of a specific compound such as 1,25-(OH)_2_-VD3 as solely the assessment of the concentration of the metabolite or a related compound (e.g. precursor). Changes in specific plasma metabolites or changes in the ratio of specific metabolites have biomarker potential especially since high fold changes were measured for specific metabolites such as a downregulation by >87-fold for plasma 2alpha-(3-Hydroxypropyl)-1alpha,25-dihydroxy-19-norvitamin D3 or downregulation by >37-fold for 1,25-dihydroxyvitamin D3 3-glycoside (Supplementary Table S1). These changes would be easier to measure and to evaluate than changes in 25-(OH)-VD3. Due to their high concentration range these compounds could potentially more reliably reflect the epileptogenic potential of an injury. Further validation of these markers is required. Finally, most VD assays only take the few major VD metabolites into account. A limitation to the development of assays including other, more complex VD metabolites as well as validation of VD metabolite biomarkers, is to some extent the availability of reference material as most metabolites are not commercially available. Considering their complex stereochemistry, custom manufacture would be costly. However, the details of VD involvement in epileptogenesis and the biomarker potential of 25-(OH)-VD3 and VD3 metabolites is an important area to study and remains to be explored in either experimental or clinical studies.

Our results also show a significant decrease in 25-(OH)-VD3 from 48 h to 12 wk in control animals; this decrease could be related to diet. The chow fed to these animals in our animal facility contained 1.5 IU/g VD3 vs. 2.0 IU/g VD3 at the vendor (Harlan). In addition to diet, plasma VD3 levels are known to decrease with age in humans^40^ which could also be the case in rats. Consistent with an age-related decline, we observed a significant decrease in VD3 levels in rats of 10–12 wk of age (48 h/acute time point) vs. 22–24 wk old (12 wk/chronic time point). Our data suggest a tight homeostatic control of VD3 metabolism such that a maximal depletion, due either to injury or aging, was reached. Nevertheless, 25-(OH)-VD3 data, assessed by the use of a targeted HPLC-MS/MS assay, support our untargeted findings; overall, this shows the potential of our untargeted methodology to investigate metabolome changes in plasma and tissue and filter out changes with relation to disease/disease pathogenesis.

There are several explanations for the lack of change of 25-(OH)-VD3 in plasma 6 h post KA-injection (ca. 4 h post SE onset). This could indicate that 1) acute SE is responsible for the decrease in 25-(OH)-VD3 plasma levels, but a longer timespan is necessary for peripheral effects to manifest, and/or that 2) acute SE itself is not responsible for the decrease in 25-(OH)-VD3 plasma levels and 25-(OH)-VD3 plasma levels decrease during epileptogenesis and are not an acute consequence of SE. Data from our cohort of rats (14 wk of age at the time of KA administration) indicates that sufficiently high levels of plasma 25-(OH)-VD3 have to be present to yield a significant decrease 48 h after SE. Furthermore, plasma 25-(OH)-VD3 concentrations of about 6 ng/mL after 12–14 wk are insufficient to yield a significant response. In conclusion, initial plasma 25-(OH)-VD3 concentrations have to be sufficient in order to yield a significant decrease in 25-(OH)-VD3 48 h and 1 wk after SE, which might be due to the epileptogenicity of the injury (SE).

Changes in lipid metabolism during KA-induced neuronal injury have been investigated in previous studies, but rarely using systems biology approaches^41^. Various studies investigated metabolic changes during or shortly after SE rather than during epileptogenesis^41^,^42^,^43^. These studies were limited to investigation of acute time points up to 72 h following chemoconvulsant-induced SE, consequently lack data from later time points. Guan *et al*. investigated changes in hippocampal lipid metabolism during KA-induced neuronal injury in an untargeted fashion at one and three days post-intraventricular KA injection; this study showed reductions in hippocampal phospholipids with mainly polyunsaturated fatty acyl chains and increases in acylated forms of phosphoethanolamines and ceramides^41^. While ceramides, including lactosyl- and glucosylceramides, were upregulated in the acute and latent phases of the present study (Fig. 2 and Supplementary Fig. S3), we did not find substantial downregulation of phospholipids in the hippocampus at 48 h, however acylglycerophosphocholines (reported as upregulated by Guan *et al*.) were decreased in plasma at this time point. Spingolipids, especially ceramides, are known to play a role in epileptogenesis^42^,^43^. Ceramide, the precursor of ceramides, induces programmed cell death in tissues and is reported to be upregulated after KA-43 and pilocarpine-induced^42^ SE which supports our results.

Increases in the precursor ceramide are attributed to NMDA receptor activation in both of these epilepsy models. In our study, many changes in ceramides were seen in the hippocampus, especially at 1 wk; some changes were seen at 12 wk in plasma samples. This, in consideration of the fact that ceramides are linked to stress responses such as cell senescence, cell cycle arrest, and apoptosis^21^, corroborates a role of ceramides in epileptogenesis and the manifestation of spontaneous seizures^21^. Excitotoxic neuronal death occurs due to excessive, prolonged receptor activation by excitatory amino acids, e.g. glutamate, which results in epileptic seizures/SE. It is a mixed form of cell death with morphologies typical of both apoptosis and necrosis^44^. Changes in ceramides in acute and chronic phases could be a result of acute seizure activity triggering apoptosis and be an indicator of disease development/progression. Changes at the latent time point could be a hallmark of disease progression/manifestation representing abnormalities in the life cycle of cells potentially promoting epileptogenesis. In contradiction with our study, a study of changes in lipid molecular species in progressive epilepsy with mental retardation by Hermansson *et al*. revealed reduction of ceramide, galactosyl- and lactosylceramide, and sulfatide in cerebral brain samples^45^. This discrepancy may be due to the fact that Hermansson *et al*. evaluated cerebrum while we studied the hippocampus. Decreases in long fatty acyl chain-containing species of the aforementioned classes were also reported by Hermansson and colleagues^45^, which is in concordance with decreases in 1-/2-acylglycerophosphocholines we found at the 48 h time point in plasma and at 48 h and 12 wk time points in the hippocampus (Supplementary Fig. S2). Galactosyl- and lactosylceramides, in our study were primarily increased at 48 h in the hippocampus and plasma, are derived directly form ceramides and are precursors for glycosphingolipids such as gangliosides. Interestingly, gangliosides were mainly downregulated in hippocampus and plasma at various time points in our study (Fig. 2). Gangliosides are essential parts of neurons and are responsible for cell signaling and cell-cell recognition^46^. Disorders of ganglioside biosynthesis are often associated with seizures and dysregulation of excitatory neurotransmission.

Ether lipids, lipids in which one or more fatty acid alkyl chains are attached to the glycerol backbone via ether instead of ester bonds, are abundant in the brain^47^. Plasmalogens, phosphoether lipids based on a glycerol backbone with the ether linkage in the sn-1 and the ester linkage in the sn-2 position, play a role in cell signaling, the immune system, and may be important as a defense mechanism against reactive oxygen species (ROS)^48^. ROS contribute mechanistically to deficits in mitochondrial respiration, which has been implicated in the etiology of TLE^2^.

The purinergic system plays a role in modulation of excitatory neurotransmission^49^. Adenosine affects pre- and postsynaptic receptors and therefore neurotransmitter release (e.g. glutamate, acetylcholine, norepinephrine, 5-hydroxytryptamine, dopamine, GABA, others)^49^. Downregulation of adenosine, the direct precursor or inosine and hypoxanthine, and concomitant upregulation of inosine and hypoxanthine at 1 wk in the hippocampus could indicate increased turnover of adenosine in the latent phase. This could be due to its neuroprotective properties as adenosine acts as an endogenous anticonvulsant agent^49^,^50^. Generally, elevated adenosine levels seem to protect areas of metabolic demand from cellular damage^49^,^50^. The fact that adenosine is decreased during the latent period in the hippocampus implies that the metabolic demand for this neuroprotective agent is unmet during this phase of epileptogenesis.

Generation of nitric oxide (NO), superoxide, and peroxynitirite are known to cause neuronal injury (lipid peroxidation, mitochondrial dysfunction, reduction in energy levels) after KA injection and are implicated in the pathogenesis of epilepsy^2^,^51^,^52^. Therefore, decreases in plasma and hippocampal citrulline levels in our study at 48 h could be linked to NO production during and after SE. NO is generated via NOS from arginine with citrulline as a coproduct, which can serve as an indirect measure of NO production^51^. Investigating the involvement of NO in KA-induced exitotoxicity in several rat brain areas, Milatovic *et al*. report increased formation of citrulline in the hippocampus 30 min after KA injection and with the onset of seizures; at 24 h, citrulline levels in the hippocampus were restored to control levels^51^. Decreased citrulline levels after 48 h in our study could be due to depletion of the precursor pool after excessive turnover of arginine to citrulline and NO during SE. In agreement with our data are results from a study involving human subjects published by Jeter *et al*.^53^. Plasma levels of citrulline and other arginine metabolites were decreased in patients 24 h after severe traumatic brain injury (TBI) compared to other study groups (healthy volunteers, patients with mild TBI, and orthopedic injury patients without TBI).

Our findings in regard to the metabolism of cholesterol and metabolites could reflect upregulated demand and metabolism of cholesterol in the brain as cholesterol is a main component of cell membranes and essential in the nervous system^54^. Increased cholesterol is consistent with upregulation of hippocampal VD3 metabolites and downregulation of 25-(OH)-VD3, eventually indicating high demand and rapid turnover of 25-(OH)-VD3 as discussed above.

Bile acids are derived from cholesterol (Fig. 5), have signaling functions, and regulate lipid, glucose, and energy metabolism^55^. They activate nuclear receptors such as the vitamin D receptor (VDR). VDR regulates calcium homeostasis, drug and bile acid metabolism, and inflammation^55^,^56^. In addition, VDR is mainly activated through 1,25-(OH)_2_-VD3 which has been shown to influence bile acid secretion^57^. Dysregulation of VD3 metabolisms, in particular 1,25-(OH)_2_-VD3, could be responsible for effects seen in bile acid concentrations (Fig. 2).

Cholesterol also is the precursor for neurosteroids such as allopregnanolone and tetrahydrodeoxycorticosterone (THDOC)^58^,^59^. Neurosteroids are broad-spectrum anticonvulsants, have antiepileptogenic properties, and are considered drug targets for epilepsy pharmacotherapy^58^. Dysregulation of neurosteroids synthesis may play a role in epileptogenesis and has been investigated in several epilepsy models^58^. Changes in THDOC (upregulated in plasma at 48 h and in the hippocampus at 1 wk) and 12alpha-methylpregna-4,9(11)-diene-3,20-dione (downregulated in the hippocampus at 1 wk) could indicate general changes in neurosteroid concentrations with possible relation to epilepsy and epileptogenesis. THDOC is a positive regulator of GABA-A receptors^59^. Increased chloride influx through GABA-A receptors hyperpolarizes the neuron and inhibits excitatory neurotransmission. SE is known to increase THDOC levels in plasma and the brain and to increase seizure threshold^59^, changes we measured at the 48 h and 1 wk time points in our study, respectively.

Diverse pathways and metabolite groups apparently involved in epileptogenesis are directly linked to one another (Fig. 5). Many of the affected pathways are mitochondrial and indicative of mitochondrial dysfunction, possibly due to oxidative stress^2^,^11^,^60^. Synthesis and metabolism of cholesterol is largely a mitochondrial process^61^,^62^ and blockage of the mevalonate pathway via which cholesterol is synthesized has been associated with mitochondrial dysfunction^61^. Furthermore, cholesterol is metabolized to its oxygenated forms, hormonal steroids, and bile acids via mitochondrial enzymes^62^. Changes in cholesterol derivatives could therefore be attributable to mitochondrial changes. Mitochondrial dysfunction is also linked to apoptosis (mitochondrial apoptotic pathway)^61^, as indicated by changes in lipids, especially ceramides. Dysregulation of carnitines suggests impaired mitochondrial beta-oxidation of lipids, which could be the result of mitochondrial damage possibly due to excessive ROS production during SE, epileptogenesis, and/or seizures.

It may be valuable to examine changes in metabolites of the VD3 pathway (e.g. previtamin D3, cholecalciferol, and 1,25-(OH)2-VD3) and their relation to epileptogenesis in future studies. Changes in primary bile acids and precursors (e.g. 7alpha-hydroxycholesterol and 7alpha-hydroxycholest-4-en-3-one) should be considered as well due to their potential to influence VD3 synthesis, as well as metabolites of the steroid hormone biosynthesis pathway (e.g. pregnenolone, pregnanolone, pregnanediol, 11-deoxycortisol, cortisol, cortisone, progesterone, corticosterone) as a dysregulation of steroid hormones has been implicated in epilepsy development and chronic epilepsy.

## Conclusions

In summary, KA-induced epilepsy development was associated with significant alterations in lipid metabolism and the metabolism of purines, cholesterol, and cholesterol-derived or -associated compounds. Many changes in pathways/metabolites during epileptogenesis could result from mitochondrial damage due to ROS either produced during SE or spontaneous seizures. Changes in VD3 metabolites in hippocampus and plasma at various time points and decreased plasma 25-(OH)-VD3 in the acute and latent phase of injury-induced epileptogenesis suggest that a panel of VD3 metabolites could serve as a biomarker of brain injury-induced epileptogenesis. Changes in VD3 and other distinct metabolites need to be validated in future studies in order to determine whether these compounds can serve as reliable markers that reflect the severity of an epileptogenic insult and the risk of epilepsy development. The translation of our results to epilepsy patients may lead to the unraveling of mechanisms involved in epilepto- and ictogenesis, and to the discovery of plasma biomarkers for both phenomena. Decreases in 25-(OH)-VD3 (acute and latent phase) in plasma and decreases in the precursor 7-dehydrocholesterol (latent and chronic phase) in the hippocampus suggest an increased turnover of these compounds to 1,25-(OH)_2_-VD3 in the hippocampus possibly due to the neuroprotective and/or immunomodulative properties of this metabolite. Significant decreases in 25-(OH)-VD3 beyond a certain level (or other VD3 metabolites/the ratio of specific VD3 metabolites to others) could be associated with the severity of a brain insult.

## Methods

### Animal Study

#### Induction of epileptogenesis, sample collection, and group size

Animal studies were approved by the Animal Care and Use Committee of the University of Colorado Denver and carried out in accordance with the approved guidelines as previously described^10^. Animals received standard care, but handling of animals prior to and during the experiment was kept to a minimum. Status epilepticus (SE) and subsequent chronic epilepsy in adult rats was induced by chemoconvulsant administration^10^. Briefly, male Sprague-Dawley rats (~300–350 g, n = 52) were subcutaneously administered sterile buffered saline, pH 7.4 (vehicle) or 11 mg/kg KA (Nanocs KA-0002) dissolved in vehicle. Animals were observed for SE (see *Monitoring of convulsive seizures* below). Mortality rate was 5–10%. Rats were sacrificed by CO_2_ inhalation and subsequent decapitation at 48 h, 1 wk, and 12 wk post-injection to encompass the acute, latent, and chronic phases of epilepsy development. The 1 wk time point was based on our previous studies and chosen to represent the latent phase when seizure probability is low. All animals were sacrificed between 9 am and noon. Plasma was collected. Hippocampi were dissected out, frozen in liquid N_2_, and stored at −80 °C until analysis. For metabolomics experiments groups comprised 3–6 animals as follows: 48 h controls n = 5, 48 h KA-treated n = 3, 1 wk controls n = 4, 1 wk KA-treated (SE, responders) n = 4, 1 wk NSE (KA-treated but no SE) n = 4, 12 wk controls n = 4, 12 wk KA-treated n = 6). For 25-(OH)-VD3 measurements after 6 h of SE and 48 h after SE in animals of 14 wk of age numbers were as follows: 6 h controls n = 4, 6 h KA-SE n = 7, 48 h controls n = 3, 48 h KA n = 5.

#### Monitoring of convulsive seizures

Behavioral seizures were evaluated by direct observation for 6 h after KA injection and scored based on a Racine scale (Racine, 1972)^10^. Only class III, IV, and V motor seizures were considered. SE, a period of continuous seizure activity, was defined as five seizures resulting in bilateral forelimb clonus and loss of balance within one hour (Racine, 1972)^10^. KA-injected animals that did not experience SE were labeled NSEs; these were sacrificed at the 1 wk time point and only included in the study for 25-(OH)-VD3 measurements. To confirm chronic epilepsy, rats were directly observed and monitored with 24/7 video for 72 h in the University of Colorado *In Vivo* Neurophysiology Core for seizure number, duration, and severity at the end of the 12 wk time point. Rats observed to have spontaneous, convulsive seizures during the monitoring period were considered chronically epileptic.

### Sample preparation

100 μL plasma or 10 mg hippocampal tissue (1:10 tissue:H_2_O with 0.1% ammonium acetate + 0.03% butylated hydroxytoluene, homogenized by sonication), respectively, were used per sample. Samples were prepared using a modified liquid-liquid extraction based on the protocol by Matyash *et al*.^63^ and Yang *et al*.^64^ for lipophilic and aqueous compounds, respectively. Briefly, protein in plasma samples was precipitated with methanol (MeOH), and supernatants were extracted with methyl tert butyl ether (MTBE) twice. Tissue samples were extracted with MeOH:MTBE 1:3 twice. Subsequent to extraction, the lipophilic fraction from plasma was resuspended in 200 μL MeOH, the lipophilic fraction from tissue in 200 μL 1:2 MeOH:chloroform, the aqueous fraction from plasma and tissue samples in 100 μL 5% acetonitrile (ACN). Blank and instrument quality control (QC) samples were prepared with each batch for sample preparation QC purposes.

### HPLC-MS analysis

Lipid fractions were analyzed on a 6210 time-of-flight mass spectrometer (TOF-MS, Agilent Technologies) and aqueous fractions on a 6510 Q-TOF-MS (Agilent Technologies). Both systems were coupled to HPLC systems (Agilent Technologies, 1200 series). Instruments were run in positive electrospray ionization (ESI) mode with scan range 50–1700 m/z and scan rate of 2 spectra/sec as described previously^64^.

HPLC parameters (aqueous): Fractions were separated at a flow rate of 0.6 mL/min. 1 μL of sample was injected onto a Phenomenex Kinetex HILIC analytical column (2.6 μm, 2.1 × 50 mm) with a ZORBAX Eclipse C8 guard cartridge (5 μm, 2.1 × 12.5 mm). Mobile phase A was 50% ACN with pH 5.8 5 mM acetic acid and mobile phase B was 90% ACN with pH 5.8 5 mM acetic acid. Gradient elution was as follows: 100% B starting point and held for 2 min, decrease to 90% B over 0.1 min, decrease to 50% B over 6.5 min, decrease to 0% B over 0.1 min, held for 6 min, increase to 100% B over 0.1 min, and held at 100% B for 9.7 min for column re-equilibration. Total run time per sample was 24.5 min.

HPLC parameters (lipids): Fractions were separated at a flow rate of 0.7 mL/min. 4 μL of sample were injected onto a Agilent Rapid Resolution SB-C18 analytical column (1.8 μm, 2.1 × 100 mm) held at 60 °C with a ZORBAX SB-C18 guard cartridge (1.8 μm, 2.1 × 5 mm). Mobile phase A was water with 0.1% formic acid and mobile phase B was 60:36:4 isopropyl alcohol:ACN:H_2_O with 0.1% formic acid. The gradient for lipid analysis was as follows: 30% B initial starting point and held for 1 min, increase to 100% B over 6 min, held for 5 min, decrease to 30% B over 1 min, and held at 30% for 4 min for column re-equilibration. To avoid carry over blanks were runs between samples with the following gradient: 30% B starting point and held for 0.5 min, increase to 100% B over 2 min, held for 0.25 min, decrease to 30% B over 0.25 min, and held at 30% for 1 min for column re-equilibration. Total run time per tissue sample was 21 min including the blank injection.

MS instrumental parameters (aqueous): gas temperature 300 °C, gas flow 10.0 L/min, nebulizer pressure 30 psi, fragmentor 120 V, ESI capillary voltage 4000 V with reference mass 121.05087 and 922.00979 (Agilent reference mix).

MS instrumental parameters (lipids): gas temperature 300 °C, gas flow 12 L/min, nebulizer pressure 30 psig, fragmentor 120, skimmer 60, octopole RF 250, capillary voltage 4000 V with reference mass 121.05087 and 922.00979 (Agilent reference mix).

### Quality Control

Samples were randomized prior to extraction and analysis. The analytical quality of methods and instruments were monitored by instrument QC samples (pooled sample) injected every 5 samples followed by a blank injection. For QC purposes, seven compounds (creatinine-d3, valine-d8, testosterone-d2, cis-10-nonadecenoic acid, N-(heptadecanoyl)-sphing-4-enine, 1,2-heptadecanoyl)-sphing-4-enine, 1,2-heptadecnoyl-sn-glycero-3-phosphotthanolamine, 1,2-heptadecanoyl-sn-glycero-3-phosphocholine) were added to plasma and tissue samples as well as QC samples as previously described^64^. Tissue samples were spiked with 5 additional compounds (Sphingosine-d7, Allopregnanolone-d4, C16:0-d31-SM, C16:0-d31-C18:1 PC, Arachidonic acid-d8). The QC internal standards were analyzed for mass accuracy within 3 ppm, retention time reproducibility and HPLC pressure curve reproducibility of less than +/−5% at 10 time points, volume under the curve, and intensity at +/−5%. % CVs for retention time and peak area were <20% for each standard regardless of fraction or matrix. Results are shown in Supplementary Table S9. Standards served QC purposes, but were not used for normalization.

### Data analysis and statistical analysis

Mass Hunter software (Agilent technologies) was used for data extraction as described previously with slight modifications^65^. The following parameters were used: Find by Molecular Feature algorithm, charge states were limited to a maximum of 2, ion species allowed were H^+^, Na^+^, K^+^, and NH_4_^+^. Mass Hunter Profinder software (Agilent technologies, version 6.0) was used with the following parameters: Batch recursive feature extraction; ion species allowed were H^+^, Na^+^, K^+^, and NH_4_^+^; charge states were limited to a maximum of 2; compound ion count threshold was set to two or more ions; EIC tolerance for mass was set to 10 ppm; for retention time to 0.2 and 0.35 min for lipid and aqueous runs, respectively; absolute height >2000 counts. Data were filtered by selection of compounds that were present in at least 50% of samples per condition. Features from sample preparation and instrument blanks were subtracted from features present in samples in order to eliminate noise. Compound lists were processed with Mass Hunter Quantitative Analysis (Agilent Technologies) software to further filter out potential false positives by removal of retention time outliers using a 0.3 min cut-off window. Mass Hunter Mass Profiler Professional (MPP; Agilent Technologies, version B.12.05) was used for differential analysis. Student’s unpaired t-tests and Benjamini-Hochberg false discovery rate multiple testing correction using asymptotic p-value computation with a corrected p-value cut-off of 0.05 were used. Fold change cut-offs of 1.5 were applied as an additional measure of QC. Compounds that were altered with a p value < 0.05 and exhibited >1.5-fold change between groups were classified as “changing” compounds unless otherwise stated in the text.

#### Annotation of metabolites and compound groups and pathway analysis

Metabolites were tentatively identified using Mass Hunter Qualitative Analysis software (Agilent Technologies) and an in-house database comprising data from the Human Metabolome Database (HMDB), Lipid Maps, and Metlin as previously described^65^. The software annotates compounds based on isotope ratios, accurate mass, chemical formulae, and scores. Elements for molecular formula generation were C, H, N, O, S, and P. A 10 ppm mass error cut-off was used with a neutral mass range up to 1700 Da and positive ions selected as H^+^, Na^+^, K^+^, and NH_4_^+^. Database identifications were limited to the 10 best matches based on score, charge state was limited to a maximum of two. All identifications are Metabolomics Standard Initiative level 2 based on the proposed minimum reporting standards^16^. If no annotation through database searches was possible, molecular formulae were generated. If no formula could be generated, compounds were represented by a compound number and their retention time. Compounds represented by a formula or compound number and retention time are referred to as “unannotated” compounds. Only annotated compounds were subjected to more specific analyses; changes in unannotated compounds are only discussed for completeness and to reflect patterns of changes. MPP was used for summarization and visualization of data. Annotated compounds and their normalized abundance values were exported to Excel 2010 (Microsoft Corporation, Redmond, WA) for visualization and organization. For organization of data in Venn diagrams, a freely available online tool provided by the University of Gent, Belgium, was used^66^.

Results are discussed in regard to changes in specific metabolites and metabolite groups as well as pathways assigned by MetaboAnalyst software. Annotated compounds were assigned to one of the 19 following groups: (1) Bile acids and bile acid metabolism intermediates, (2) Carbohydrates and sugars, (3) Carnitines, (4) Ceramides, glucosylceramides, and ceramide phosphoinositols, (5) Cholesterol, cholesterol esters, and intermediates, (6) Diacylglycerols, (7) Gangliosides, (8) Nucleosides, nucleotides, purine, and pyrimidine metabolism (9) Organic acids and derivatives, (10) Other, (11) Peptides, (12) Phosphatidic acids, (13) Phosphatidylcholines, (14) Phosphatidylethanolamines and phosphatidylceramides, (15) Phosphatidylinositols, (16) Phosphatidylserines, (17) Sphingomyelins, (18) Triacylglycerols, or (19) Vitamin D and derivatives. Categories were assigned dependent on HMDB super classes, classes, and subclasses or manually in case no HMDB entry was available (e.g. vitamin D metabolites). Certain categories were specified further dependent on the nature of the metabolites to enhance conciseness of designations, e.g. carnitines and phosphatidylcholines. Metabolites listed under “Other” (10) belong to the following categories with less than 3 compounds per category: amines (Palmitoleoyl-EA), aldehydes (4-aminobutyraldehyde), steroids and steroid derivates (12alpha-methylpregna-4,9(11)-diene-3,20-dione, tetrahydrodeoxycorticosterone), monoglycerols (MG(18:0e/0:0/0:0), MG(20:2(11Z,14Z)/0:0/0:0)), glycerophosphoglycerols (PG(22:6(4Z,7Z,10Z,13Z,16Z,19Z)/22:6(4Z,7Z,10Z,13Z,16Z,19Z))), metabolites of sphingosine (N,N-dimethylsphingosine), phytosterols (plant-derived, present in small amounts in humans and animals; 22:0-Glc-stigmasterol, 22:2-Glc-stigmasterol), quaternary ammonium salts/alkaloids and derivatives (Neurine), phosphatidylglycerolphosphate (PGP(18:1(11Z)/20:3(8Z,11Z,14Z)), PGP(18:1(11Z)/22:5(7Z,10Z,13Z,16Z,19Z))), intermediates in fatty acid metabolism (2E-tetradecenoyl-CoA), fatty amides/N-acyl amides ((R)-(16,16-dimethyldocosa-cis-5,8,11,14-tetraenoyl)-1′-hydroxy-2′-propylamine). Pathway enrichment analysis was conducted based on KEGG identifiers using MetaboAnalyst^19^. For pathway coverage compounds with KEGG ID C00107 (Dipeptide) were marked as C00012 (Peptide). Pathway analysis showed significance for selected pathways only; discussed are significantly changed pathways as well as pathways with possible relation to epileptogenesis despite their p-values > 0.05 (not significantly changed).

### HPLC-MS/MS analysis of plasma 25-(OH)-VD3

Plasma levels of 25-(OH)-VD3 were measured by one of two established clinical assays at the Pharmacokinetics Laboratory at National Jewish Health (Denver, CO; initial cohort of animals, i.e. plasma of acute, latent, and chronic animals that were also subjected to metabolomics analysis) or Animal Reference Pathology (Salt Lake City, UT; second cohort of animals, i.e. samples collected after 6 h of SE and animals 14 wk of age). Assay conducted at National Jewish Health: A calibration curve of 25-(OH)-VD3 was prepared (range: 1–100 ng/mL). 40 μL of 50 pg/μL 25-(OH)-VD3-d3 as internal standard in MeOH were used per sample. Protein was precipitated and supernatants diluted. A Phenomenex Strata-X (30 mg/well) 96 well plate was used to extract analytes. Samples were dried in a speed vac and reconstituted in 60:40 MeOH/H_2_O. Samples were analyzed on an Agilent 6410 Triple Quadrupole Tandem Mass Spectrometer coupled with an Agilent 1290 Infinity HPLC system using H_2_O + 0.1% formic acid (mobile phase A) and MeOH + 0.1% formic acid (mobile phase B), with the following gradient: 0–1 min 80% B, 1–3.1 min 80–100% B, 3.1–4.6 min 100% B, 4.6–4.7 min 100–80% B, 4.7–5.5 min 80% B. Assay conducted at Animal Reference Pathology: Analysis was performed according to Kushner *et al*.^67^. Briefly, the samples were filtered in a 96-well collection plate and analyzed using an Agilent Technologies 1260 liquid chromatography system for two-dimensional chromatographic separation and ABSCIEX API 5500 LC-MS/MS detection. Statistical differences within this data set were determined by one-way ANOVA combined with Tukey’s *post hoc* test.

## Additional Information

**How to cite this article**: Heischmann, S. *et al*. Exploratory Metabolomics Profiling in the Kainic Acid Rat Model Reveals Depletion of 25-Hydroxyvitamin D3 during Epileptogenesis. *Sci. Rep.* **6**, 31424; doi: 10.1038/srep31424 (2016).

## Supplementary Material

Supplementary Information

Supplementary Table S1

Supplementary Table S2

Supplementary Table S3

Supplementary Table S4

Supplementary Table S5

Supplementary Table S6

Supplementary Table S7

Supplementary Table S9
