# Supplementary material for: Exploratory Metabolomics Profiling in the Kainic Acid Rat Model Reveals Depletion of 25-Hydroxyvitamin D3 during Epileptogenesis
Source: Sci Rep. 2016 Aug 16;6:31424. doi: 10.1038/srep31424 (PMC4985632; doi:10.1038/srep31424)
Supplement: Supplementary Information [file srep31424-s1.doc]

**Exploratory Metabolomics Profiling in the Kainic Acid Rat Model Reveals Depletion of 25-Hydroxyvitamin D3 during Epileptogenesis**

Svenja Heischmann, Kevin Quinn, Charmion Cruickshank-Quinn, Li Ping Liang, Rick Reisdorph, Nichole Reisdorph, Manisha Patel

**Supplementary Materials**

**Supplementary Figure S1.**

**Venn diagrams showing the overlap and uniqueness of changes between different matrices and time points.** (a) Venn diagram on annotated metabolites in the hippocampus. (b) Venn diagram on annotated metabolites in plasma. (c) Venn diagrams on annotated metabolites in the hippocampus and plasma. (d) Venn diagram on unannotated metabolites in the hippocampus. (e) Venn diagram on unannotated metabolites in plasma. (f) Venn diagrams on unannotated metabolites in the hippocampus and plasma. Numbers represent counts of changing metabolites (up- or downregulated).


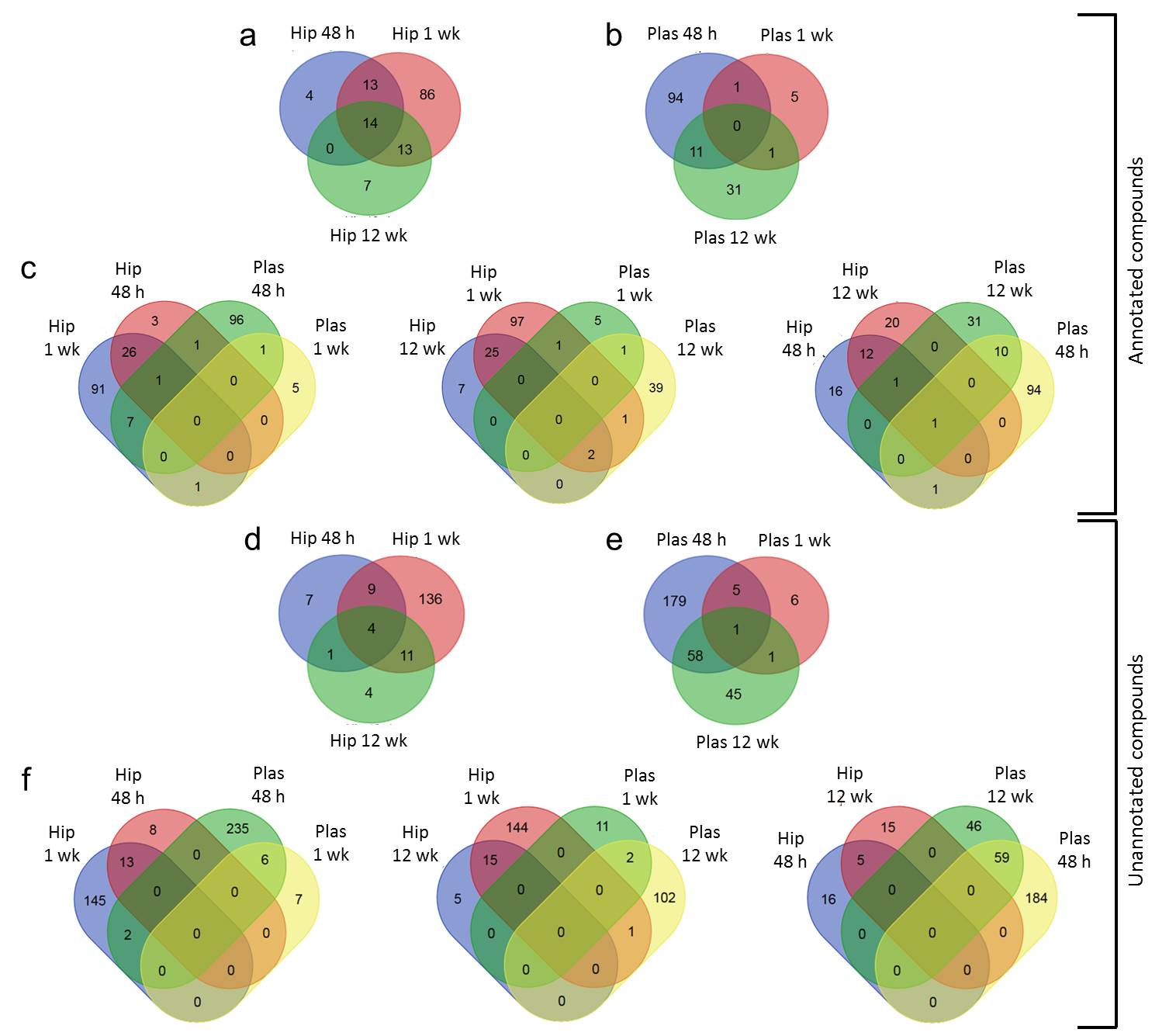


**Pathway Analysis by Metabo Analyst**

**Supplementary Table S8.**

Metabolic pathways matched by MetaboAnalyst 3.0 (<http://www.metaboanalyst.ca/MetaboAnalyst/>). Pathways are sorted according to p-values from pathway enrichment analysis and pathway impact values from pathway topology analysis for changed metabolites (p<0.05, >1.5-fold change) in hippocampus and plasma (combined) at the 48 h, 1 wk, and 12 wk time point individually and all 3 time points combined. Analysis was conducted based on KEGG IDs using the Rattus Norvegicus library containing 81 pathways, hypergeometric test for Overrepresentation Analysis (default), and Relative-betweeness Centrality for Pathway Topology Analysis (default).

------

| **Pathway Name** | **Total** | **Hits** | **p** | **-log(p)** | **Holm p** | **FDR** | **Impact** | **Details** |
| --- | --- | --- | --- | --- | --- | --- | --- | --- |
| **48h (Hippocampus and Plasma combined)**  ------ | | | | | | | | |
| [Glycerophospholipid metabolism](http://www.metaboanalyst.ca/MetaboAnalyst/faces/Secure/pathway/ResultView.xhtml) | 30 | 8 | 3.7743E-8 | 17.092 | 3.0572E-6 | 3.0572E-6 | 0.49722 | [KEGG](http://www.genome.jp/kegg-bin/show_pathway?rno00564) |
| [Sphingolipid metabolism](http://www.metaboanalyst.ca/MetaboAnalyst/faces/Secure/pathway/ResultView.xhtml) | 21 | 4 | 6.0432E-4 | 7.4114 | 0.048346 | 0.024475 | 0.41604 | [KEGG](http://www.genome.jp/kegg-bin/show_pathway?rno00600) |
| [Glycosylphosphatidylinositol (GPI)-anchor biosynthesis](http://www.metaboanalyst.ca/MetaboAnalyst/faces/Secure/pathway/ResultView.xhtml) | 14 | 2 | 0.030188 | 3.5003 | 1.0 | 0.81507 | 0.0439 | [KEGG](http://www.genome.jp/kegg-bin/show_pathway?rno00563) |
| [Glycerolipid metabolism](http://www.metaboanalyst.ca/MetaboAnalyst/faces/Secure/pathway/ResultView.xhtml) | 18 | 2 | 0.048323 | 3.0298 | 1.0 | 0.8859 | 0.12624 | [KEGG](http://www.genome.jp/kegg-bin/show_pathway?rno00561) |
| [Arginine and proline metabolism](http://www.metaboanalyst.ca/MetaboAnalyst/faces/Secure/pathway/ResultView.xhtml) | 44 | 3 | 0.054685 | 2.9062 | 1.0 | 0.8859 | 0.09442 | [KEGG](http://www.genome.jp/kegg-bin/show_pathway?rno00330) |
| [Steroid biosynthesis](http://www.metaboanalyst.ca/MetaboAnalyst/faces/Secure/pathway/ResultView.xhtml) | 35 | 2 | 0.15293 | 1.8778 | 1.0 | 1.0 | 0.0 | [KEGG](http://www.genome.jp/kegg-bin/show_pathway?rno00100) |
| **1wk (Hippocampus and Plasma combined)** | | | | | | | | |
| [Sphingolipid metabolism](http://www.metaboanalyst.ca/MetaboAnalyst/faces/Secure/pathway/ResultView.xhtml) | 21 | 5 | 2.1317E-4 | 8.4534 | 0.017267 | 0.013692 | 0.32331 | [KEGG](http://www.genome.jp/kegg-bin/show_pathway?rno00600) |
| [Ether lipid metabolism](http://www.metaboanalyst.ca/MetaboAnalyst/faces/Secure/pathway/ResultView.xhtml) | 13 | 4 | 3.3808E-4 | 7.9922 | 0.027046 | 0.013692 | 0.71429 | [KEGG](http://www.genome.jp/kegg-bin/show_pathway?rno00565) |
| [Glycerophospholipid metabolism](http://www.metaboanalyst.ca/MetaboAnalyst/faces/Secure/pathway/ResultView.xhtml) | 30 | 5 | 0.0012348 | 6.6968 | 0.097551 | 0.03334 | 0.29969 | [KEGG](http://www.genome.jp/kegg-bin/show_pathway?rno00564) |
| [Glycerolipid metabolism](http://www.metaboanalyst.ca/MetaboAnalyst/faces/Secure/pathway/ResultView.xhtml) | 18 | 3 | 0.013047 | 4.3392 | 1.0 | 0.25451 | 0.22746 | [KEGG](http://www.genome.jp/kegg-bin/show_pathway?rno00561) |
| [Steroid biosynthesis](http://www.metaboanalyst.ca/MetaboAnalyst/faces/Secure/pathway/ResultView.xhtml) | 35 | 4 | 0.01571 | 4.1534 | 1.0 | 0.25451 | 0.13278 | [KEGG](http://www.genome.jp/kegg-bin/show_pathway?rno00100) |
| [Glycosylphosphatidylinositol (GPI)-anchor biosynthesis](http://www.metaboanalyst.ca/MetaboAnalyst/faces/Secure/pathway/ResultView.xhtml) | 14 | 2 | 0.05818 | 2.8442 | 1.0 | 0.78543 | 0.0439 | [KEGG](http://www.genome.jp/kegg-bin/show_pathway?rno00563) |
| [Arginine and proline metabolism](http://www.metaboanalyst.ca/MetaboAnalyst/faces/Secure/pathway/ResultView.xhtml) | 44 | 3 | 0.12746 | 2.0599 | 1.0 | 1.0 | 0.08733 | [KEGG](http://www.genome.jp/kegg-bin/show_pathway?rno00330) |
| [Purine metabolism](http://www.metaboanalyst.ca/MetaboAnalyst/faces/Secure/pathway/ResultView.xhtml) | 68 | 3 | 0.30619 | 1.1835 | 1.0 | 1.0 | 0.01426 | [KEGG](http://www.genome.jp/kegg-bin/show_pathway?rno00230) |
| [Fatty acid metabolism](http://www.metaboanalyst.ca/MetaboAnalyst/faces/Secure/pathway/ResultView.xhtml) | 39 | 2 | 0.30662 | 1.1822 | 1.0 | 1.0 | 0.00134 | [KEGG](http://www.genome.jp/kegg-bin/show_pathway?rno00071) |
| **12wk (Hippocampus and Plasma combined)** | | | | | | | | |
| [Glycerophospholipid metabolism](http://www.metaboanalyst.ca/MetaboAnalyst/faces/Secure/pathway/ResultView.xhtml) | 30 | 8 | 2.7346E-8 | 17.415 | 2.215E-6 | 2.215E-6 | 0.34413 | [KEGG](http://www.genome.jp/kegg-bin/show_pathway?rno00564) |
| [Sphingolipid metabolism](http://www.metaboanalyst.ca/MetaboAnalyst/faces/Secure/pathway/ResultView.xhtml) | 21 | 3 | 0.0067276 | 5.0015 | 0.53821 | 0.27247 | 0.32331 | [KEGG](http://www.genome.jp/kegg-bin/show_pathway?rno00600) |
| [Ether lipid metabolism](http://www.metaboanalyst.ca/MetaboAnalyst/faces/Secure/pathway/ResultView.xhtml) | 13 | 2 | 0.024452 | 3.711 | 1.0 | 0.57088 | 0.39286 | [KEGG](http://www.genome.jp/kegg-bin/show_pathway?rno00565) |
| [Glycosylphosphatidylinositol(GPI)-anchor biosynthesis](http://www.metaboanalyst.ca/MetaboAnalyst/faces/Secure/pathway/ResultView.xhtml) | 14 | 2 | 0.028192 | 3.5687 | 1.0 | 0.57088 | 0.0439 | [KEGG](http://www.genome.jp/kegg-bin/show_pathway?rno00563) |
| [Glycerolipid metabolism](http://www.metaboanalyst.ca/MetaboAnalyst/faces/Secure/pathway/ResultView.xhtml) | 18 | 2 | 0.045212 | 3.0964 | 1.0 | 0.73244 | 0.10704 | [KEGG](http://www.genome.jp/kegg-bin/show_pathway?rno00561) |
| [Steroid biosynthesis](http://www.metaboanalyst.ca/MetaboAnalyst/faces/Secure/pathway/ResultView.xhtml) | 35 | 2 | 0.14418 | 1.9367 | 1.0 | 1.0 | 0.07884 | [KEGG](http://www.genome.jp/kegg-bin/show_pathway?rno00100) |
| [Fatty acid metabolism](http://www.metaboanalyst.ca/MetaboAnalyst/faces/Secure/pathway/ResultView.xhtml) | 39 | 2 | 0.17147 | 1.7633 | 1.0 | 1.0 | 0.00201 | [KEGG](http://www.genome.jp/kegg-bin/show_pathway?rno00071) |
| **48h, 1wk, and 12wk (Hippocampus and Plasma combined)** | | | | | | | | |
| [Glycerophospholipid metabolism](http://www.metaboanalyst.ca/MetaboAnalyst/faces/Secure/pathway/ResultView.xhtml) | 30 | 9 | 3.6214E-7 | 14.831 | 2.9334E-5 | 2.9334E-5 | 0.54166 | [KEGG](http://www.genome.jp/kegg-bin/show_pathway?rno00564) |
| [Sphingolipid metabolism](http://www.metaboanalyst.ca/MetaboAnalyst/faces/Secure/pathway/ResultView.xhtml) | 21 | 6 | 5.479E-5 | 9.812 | 0.0043832 | 0.002219 | 0.45865 | [KEGG](http://www.genome.jp/kegg-bin/show_pathway?rno00600) |
| [Ether lipid metabolism](http://www.metaboanalyst.ca/MetaboAnalyst/faces/Secure/pathway/ResultView.xhtml) | 13 | 4 | 8.0844E-4 | 7.1204 | 0.063867 | 0.021828 | 0.71429 | [KEGG](http://www.genome.jp/kegg-bin/show_pathway?rno00565) |
| [Glycerolipid metabolism](http://www.metaboanalyst.ca/MetaboAnalyst/faces/Secure/pathway/ResultView.xhtml) | 18 | 4 | 0.0030305 | 5.799 | 0.23638 | 0.061367 | 0.24666 | [KEGG](http://www.genome.jp/kegg-bin/show_pathway?rno00561) |
| [Steroid biosynthesis](http://www.metaboanalyst.ca/MetaboAnalyst/faces/Secure/pathway/ResultView.xhtml) | 35 | 4 | 0.033163 | 3.4063 | 1.0 | 0.53724 | 0.13278 | [KEGG](http://www.genome.jp/kegg-bin/show_pathway?rno00100) |
| [Arginine and proline metabolism](http://www.metaboanalyst.ca/MetaboAnalyst/faces/Secure/pathway/ResultView.xhtml) | 44 | 4 | 0.068036 | 2.6877 | 1.0 | 0.91848 | 0.11775 | [KEGG](http://www.genome.jp/kegg-bin/show_pathway?rno00330) |
| [Glycosylphosphatidylinositol (GPI)-anchor biosynthesis](http://www.metaboanalyst.ca/MetaboAnalyst/faces/Secure/pathway/ResultView.xhtml) | 14 | 2 | 0.086331 | 2.4496 | 1.0 | 0.99898 | 0.0439 | [KEGG](http://www.genome.jp/kegg-bin/show_pathway?rno00563) |
| [Fatty acid metabolism](http://www.metaboanalyst.ca/MetaboAnalyst/faces/Secure/pathway/ResultView.xhtml) | 39 | 3 | 0.15934 | 1.8367 | 1.0 | 1.0 | 0.00201 | [KEGG](http://www.genome.jp/kegg-bin/show_pathway?rno00071) |
| [Purine metabolism](http://www.metaboanalyst.ca/MetaboAnalyst/faces/Secure/pathway/ResultView.xhtml) | 68 | 3 | 0.44139 | 0.81782 | 1.0 | 1.0 | 0.01426 | [KEGG](http://www.genome.jp/kegg-bin/show_pathway?rno00230) |
| [Primary bile acid biosynthesis](http://www.metaboanalyst.ca/MetaboAnalyst/faces/Secure/pathway/ResultView.xhtml) | 46 | 2 | 0.49503 | 0.70314 | 1.0 | 1.0 | 0.03698 | [KEGG](http://www.genome.jp/kegg-bin/show_pathway?rno00120) |
|  |  |  |  |  |  |  |  |  |
|  |  |  |  |  |  |  |  |  |

**Supplementary Figure S2.**

**Changes in glycerophospholipid metabolism.** Metabolites are represented by their KEGG IDs. Metabolites that were significantly changed at any examined time point in hippocampus or plasma are marked in red. Heat maps below the pathway map show the time point and number of metabolites of a certain KEGG ID that were up- and/or downregulated in hippocampus and/or plasma.
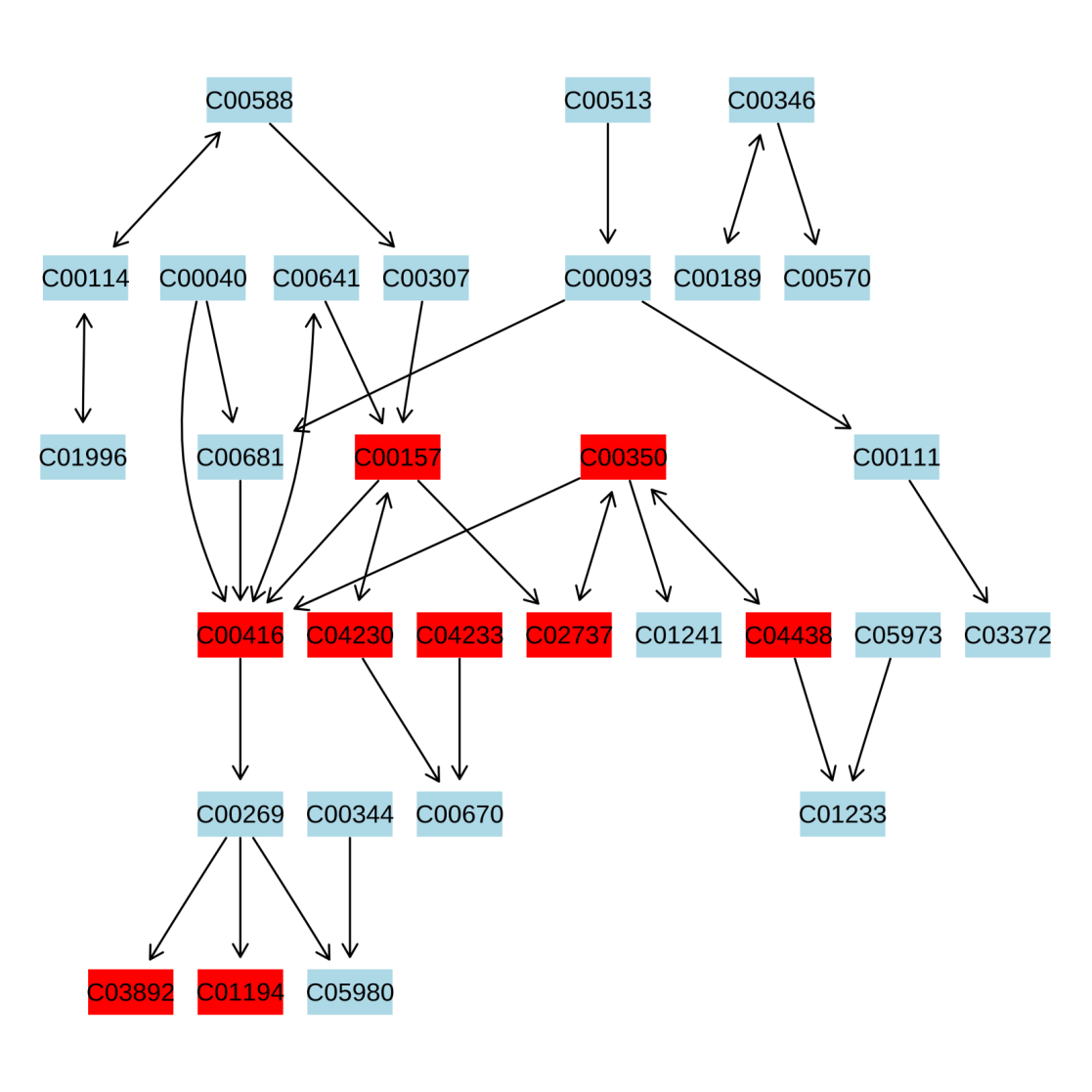


|  | **Hippocampus** | | | | | |  | **Plasma** | | | | | |
| --- | --- | --- | --- | --- | --- | --- | --- | --- | --- | --- | --- | --- | --- |
|  | **48h** | | **1wk** | | **12wk** | |  | **48h** | | **1wk** | | **12wk** | |
| **C00157: Phosphatidylcholine** | 0 | 0 | -4 | 2 | 0 | 1 |  | -2 | 2 | 0 | 1 | 0 | 1 |
| **C00350: Phosphatidylethanolamine** | 0 | 0 | 0 | 1 | 0 | 0 |  | -1 | 0 | 0 | 0 | 0 | 2 |
| **C00416: Phosphatidic acid** | 0 | 0 | 0 | 0 | 0 | 0 |  | -1 | 0 | 0 | 0 | 0 | 0 |
| **C04230: 1-Acylglycerophosphocholine** | 0 | 0 | -1 | 3 | 0 | 0 |  | -18 | 0 | 0 | 0 | -7 | 0 |
| **C04233: 2-Acylglycerophosphocholine** | 0 | 0 | 0 | 0 | 0 | 0 |  | -2 | 0 | 0 | 0 | -1 | 0 |
| **C02737: Phosphatidylserine** | -1 | 0 | -1 | 2 | 0 | 1 |  | -3 | 0 | 0 | 0 | -1 | 2 |
| **C04438: 1-Acyl-sn-glycero-3-PE** | 0 | 0 | 0 | 0 | -1 | 0 |  | 0 | 0 | 0 | 0 | 0 | 0 |
| **C03892: Phosphatidylglycerophosphate** | 0 | 0 | 0 | 0 | 0 | 0 |  | 0 | 1 | 0 | 0 | -1 | 0 |
| **C01194: Phosphatidylinositol** | 0 | 0 | -2 | 0 | -1 | 0 |  | -3 | 1 | 0 | 0 | 0 | 0 |

**Supplementary Figure S3.**

**Changes in sphingolipid metabolism.** Metabolites are represented by their KEGG IDs. Metabolites that were significantly changed at any examined time point in hippocampus or plasma are marked in red. Heat maps below the pathway map show the time point and number of metabolites of a certain KEGG ID that were up- and/or downregulated in hippocampus and/or plasma.


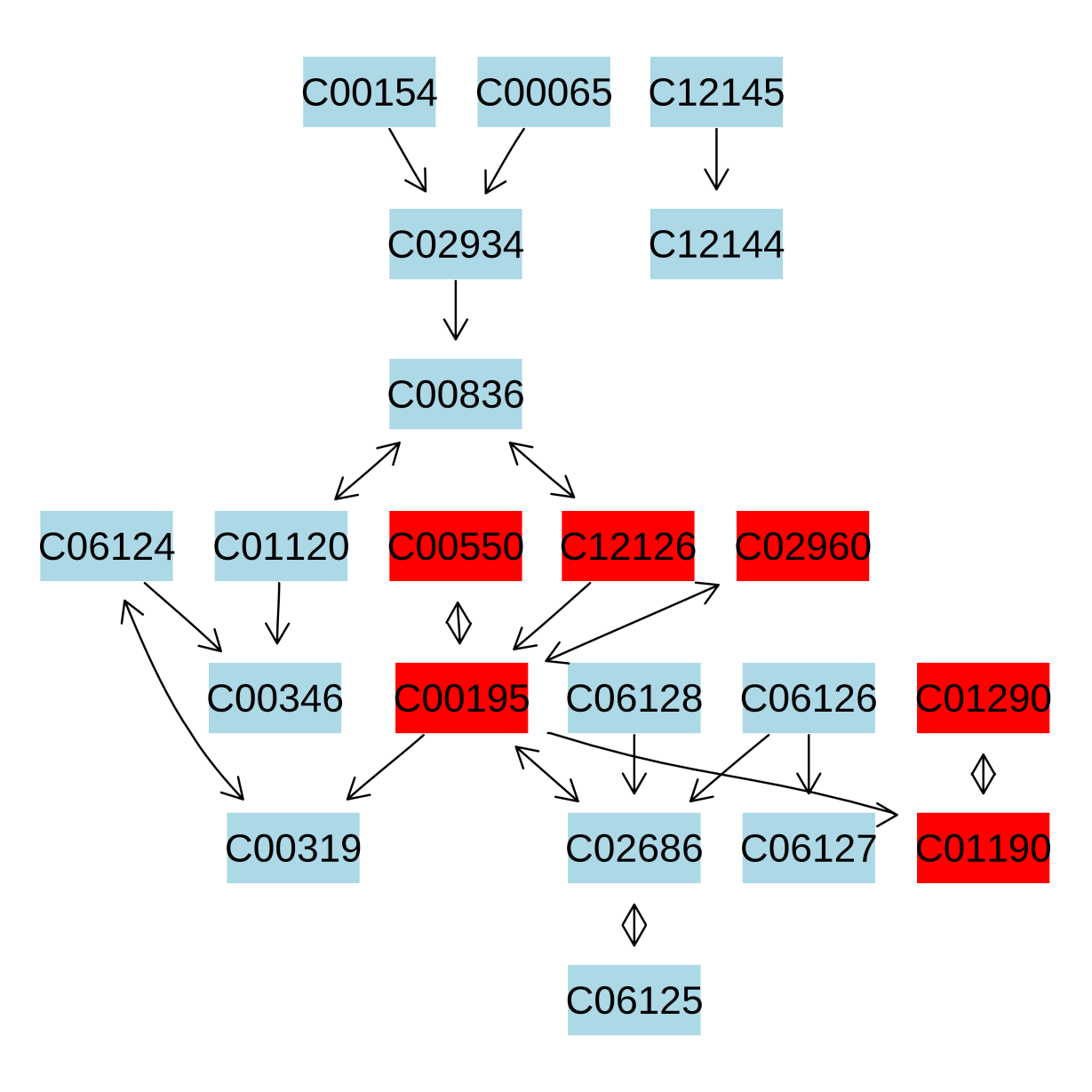


|  | **Hippocampus** | | | | | |  | **Plasma** | | | | | |
| --- | --- | --- | --- | --- | --- | --- | --- | --- | --- | --- | --- | --- | --- |
|  | **48h** | | **1wk** | | **12wk** | |  | **48h** | | **1wk** | | **12wk** | |
| **C00550: Sphingomyelin** | 0 | 0 | -1 | 1 | 0 | 0 |  | 0 | -1 | 0 | 0 | 0 | 0 |
| **C12126: Dihydroceramide** | 0 | 0 | 0 | 0 | 0 | 0 |  | 0 | -2 | 0 | 0 | 0 | 0 |
| **C02960: Ceramide phosphate** | 0 | 0 | 0 | 1 | 0 | 0 |  | 0 | 0 | 0 | 0 | 0 | 0 |
| **C00195: Ceramide** | 0 | 4 | 0 | 9 | 0 | 4 |  | 0 | 0 | 0 | 0 | 0 | 3 |
| **C01290: Lactosylceramide** | 0 | 1 | -1 | 3 | 0 | 1 |  | 0 | 0 | 0 | 0 | 0 | 0 |
| **C01190: Glucosylceramide** | 0 | 0 | 0 | 2 | 0 | 0 |  | 0 | 0 | 0 | 3 | 0 | 1 |

**Supplementary Figure S4.**

**Changes in ether lipid metabolism.** Metabolites are represented by their KEGG IDs. Metabolites that were significantly changed at any examined time point in hippocampus or plasma are marked in red. Heat maps below the pathway map show the time point and number of metabolites of a certain KEGG ID that were up- and/or downregulated in hippocampus and/or plasma.
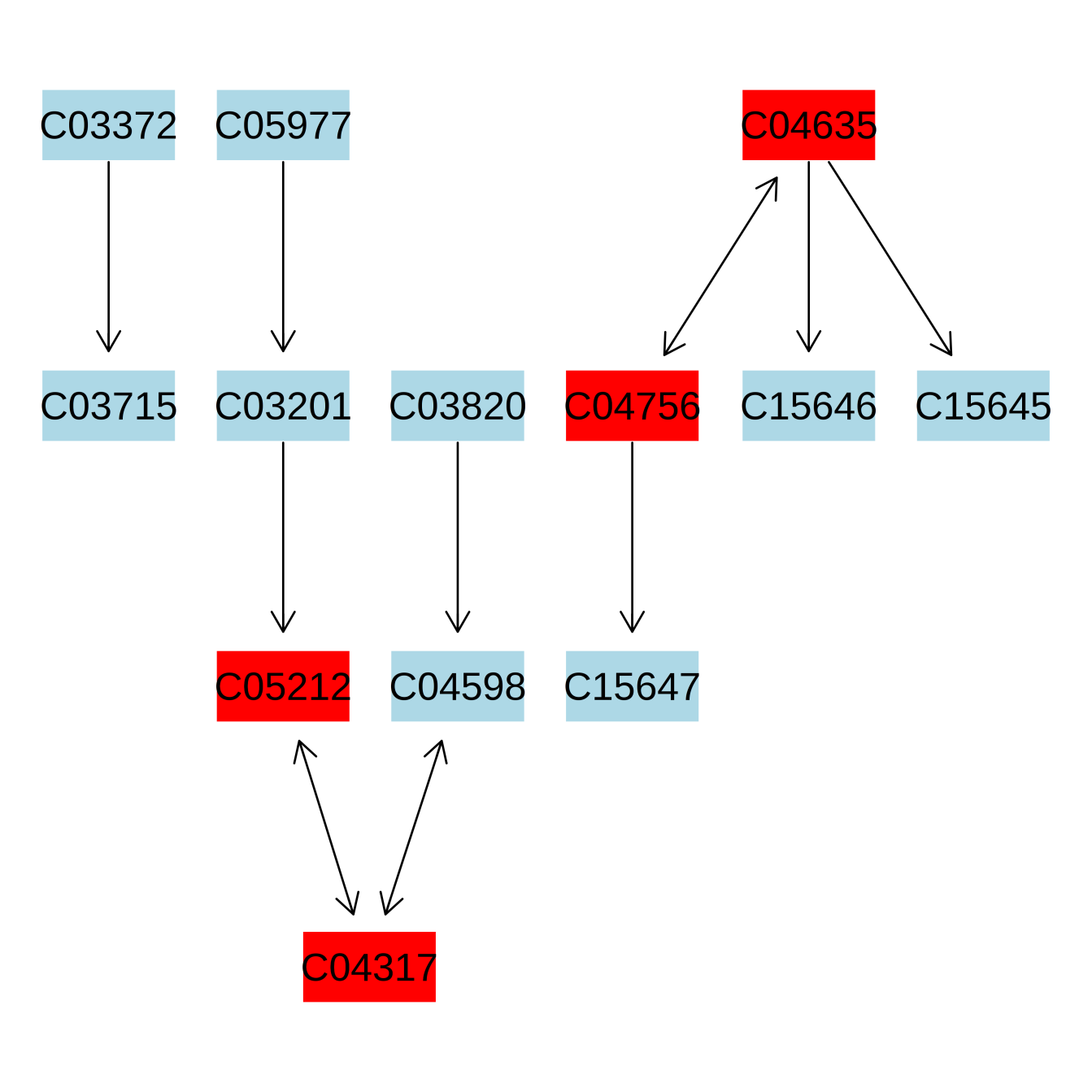


|  | **Hippocampus** | | | | | |  | **Plasma** | | | | | |
| --- | --- | --- | --- | --- | --- | --- | --- | --- | --- | --- | --- | --- | --- |
|  | **48h** | | **1wk** | | **12wk** | |  | **48h** | | **1wk** | | **12wk** | |
| **C04635: 1-Alkenylglycero-PE** | 0 | 0 | 0 | 1 | 0 | 1 |  | 0 | 0 | 0 | 0 | 0 | 0 |
| **C04756: Plasmenylethanolamine** | 0 | 0 | 0 | 1 | 0 | 0 |  | 0 | 0 | 0 | 0 | 0 | 0 |
| **C05212: 2-Acyl-1-alkyl-sn-glycero-3-PC** | 0 | 2 | 0 | 5 | 0 | 0 |  | -4 | 0 | 0 | 0 | -1 | 0 |
| **C04317: 1-Alkyl-sn-glycero-3-PC** | 0 | 0 | 0 | 1 | 0 | 0 |  | 0 | 0 | 0 | 0 | 0 | 0 |

**Supplementary Figure S5.**

**Changes in glycerolipid metabolism.** Metabolites are represented by their KEGG IDs. Metabolites that were significantly changed at any examined time point in hippocampus or plasma are marked in red. Heat maps below the pathway map show the time point and number of metabolites of a certain KEGG ID that were up- and/or downregulated in hippocampus and/or plasma.
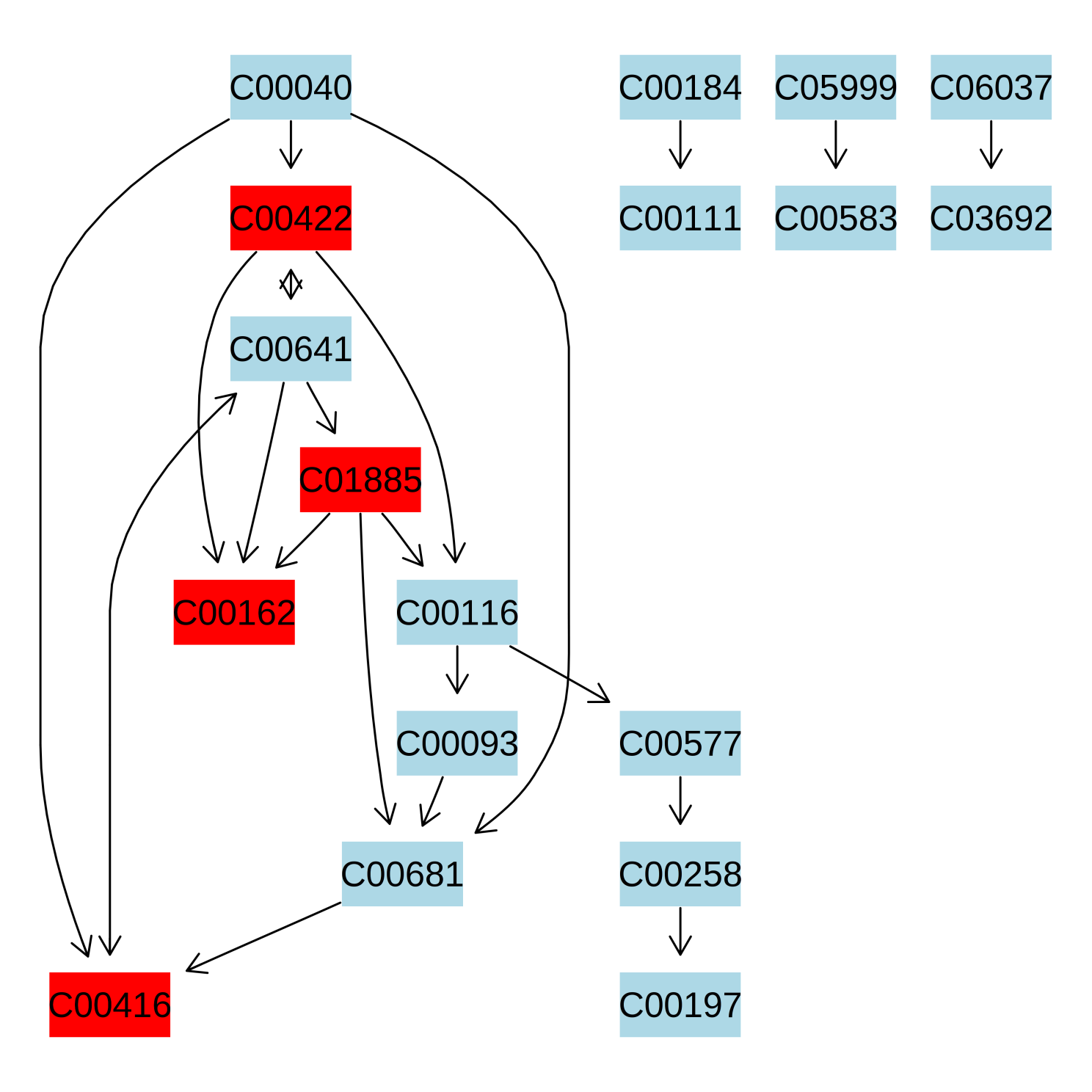


|  | **Hippocampus** | | | | | |  | **Plasma** | | | | | |
| --- | --- | --- | --- | --- | --- | --- | --- | --- | --- | --- | --- | --- | --- |
|  | **48h** | | **1wk** | | **12wk** | |  | **48h** | | **1wk** | | **12wk** | |
| **C00422: Triacylglycerol** | 0 | 1 | -1 | 4 | -1 | 0 |  | -15 | 3 | -1 | 0 | 0 | 2 |
| **C01885: 1-Monoacylglycerol** | 0 | 0 | 0 | 1 | 0 | 0 |  | 0 | 0 | 0 | 0 | 0 | 0 |
| **C00162: Fatty acid** | 0 | 0 | 0 | 1 | -1 | 0 |  | 0 | 0 | 0 | 0 | 0 | 0 |
| **C00416: Phosphatidic acid** | 0 | 0 | 0 | 0 | 0 | 0 |  | -1 | 0 | 0 | 0 | 0 | 0 |

**Supplementary Figure S6.**

**Changes in purine metabolism.** Metabolites are represented by their KEGG IDs. Metabolites that were significantly changed at any examined time point in hippocampus or plasma are marked in red. Heat maps below the pathway map show the time point and number of metabolites of a certain KEGG ID that were up- and/or downregulated in hippocampus and/or plasma.


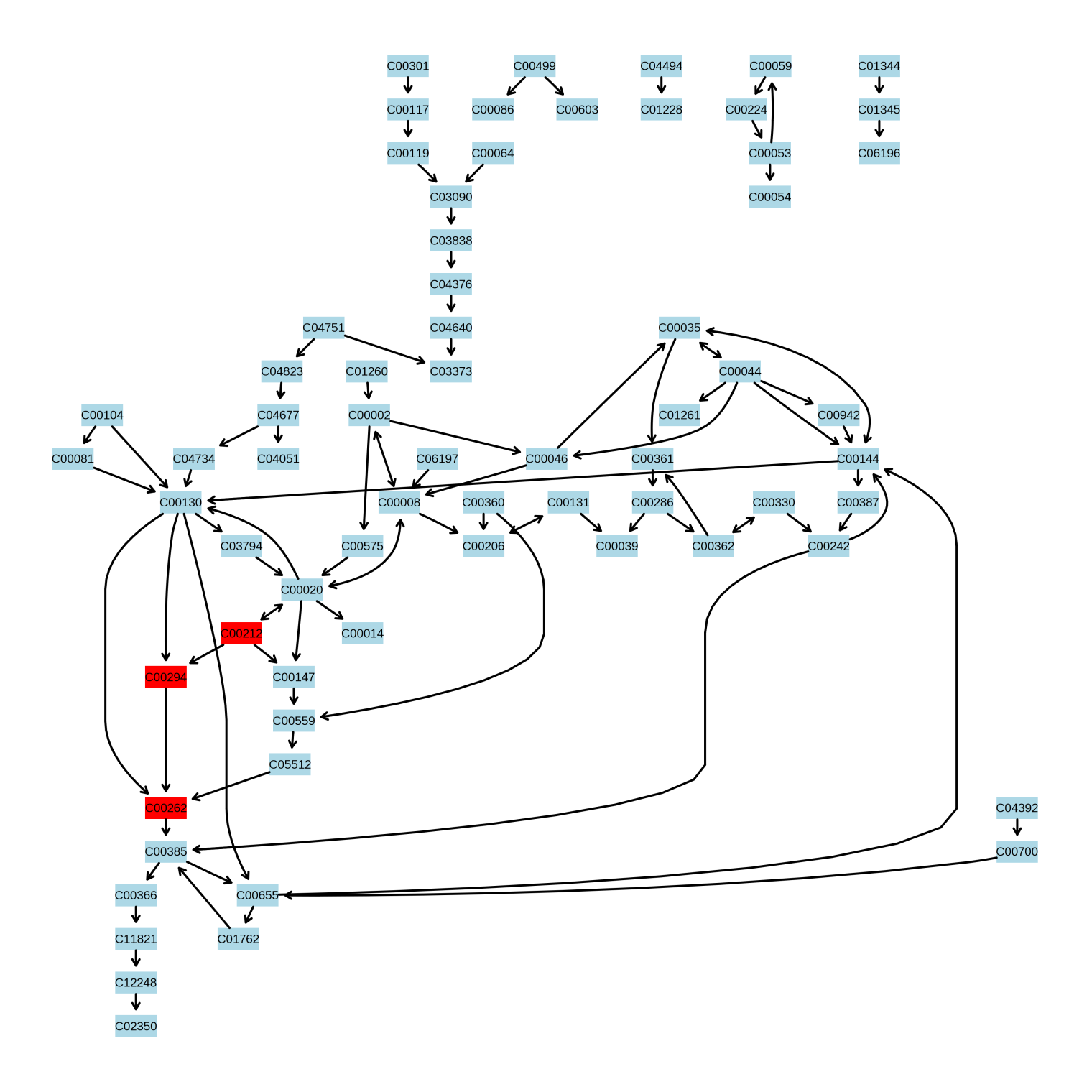


|  | **Hippocampus** | | | | | |  | **Plasma** | | | | | |
| --- | --- | --- | --- | --- | --- | --- | --- | --- | --- | --- | --- | --- | --- |
|  | **48h** | | **1wk** | | **12wk** | |  | **48h** | | **1wk** | | **12wk** | |
| **C00212: Adenosine** | 0 | 0 | -1 | 0 | 0 | 0 |  | 0 | 0 | 0 | 0 | 0 | 0 |
| **C00294: Inosine** | 0 | 0 | 0 | 1 | 0 | 0 |  | 0 | 0 | 0 | 0 | 0 | 0 |
| **C00262: Hypoxanthine** | -1 | 0 | 0 | 1 | 0 | 1 |  | -1 | 0 | 0 | 0 | 0 | 1 |

**Supplementary Figure S7.**

**Changes in arginine and proline metabolism.** Metabolites are represented by their KEGG IDs. Metabolites that were significantly changed at any examined time point in hippocampus or plasma are marked in red. Heat maps below the pathway map show the time point and number of metabolites of a certain KEGG ID that were up- and/or downregulated in hippocampus and/or plasma.
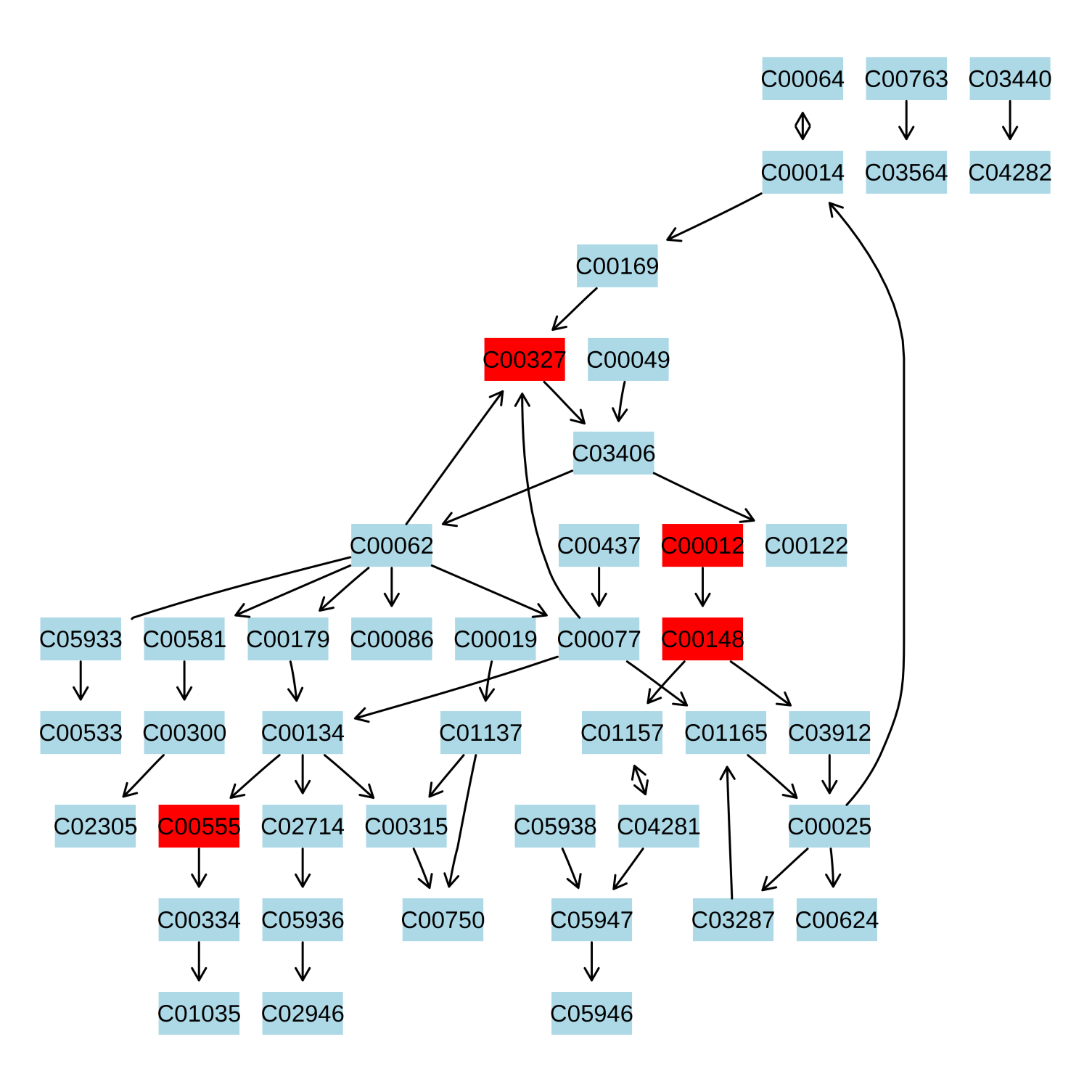


|  | **Hippocampus** | | | | | |  | **Plasma** | | | | | |
| --- | --- | --- | --- | --- | --- | --- | --- | --- | --- | --- | --- | --- | --- |
|  | **48h** | | **1wk** | | **12wk** | |  | **48h** | | **1wk** | | **12wk** | |
| **C00327: L-Citrulline** | -1 | 0 | 0 | 0 | 0 | 0 |  | -1 | 0 | 0 | 0 | 0 | 0 |
| **C00012: Peptide** | 0 | 0 | -2 | 0 | 0 | 0 |  | -2 | 0 | 0 | 1 | -1 | 1 |
| **C00148: L-Proline** | 0 | 0 | 0 | 1 | 0 | 0 |  | -1 | 0 | 0 | 0 | 0 | 0 |
| **C00555: 4-Aminobutyraldehyde** | 0 | 0 | -1 | 0 | 0 | 0 |  | 0 | 0 | 0 | 0 | 0 | 0 |

**Supplementary Figure S8.**

**Changes in steroid biosynthesis.** Metabolites are represented by their KEGG IDs. Metabolites that were significantly changed at any examined time point in hippocampus or plasma are marked in red. Heat maps below the pathway map show the time point and number of metabolites of a certain KEGG ID that were up- and/or downregulated in hippocampus and/or plasma.
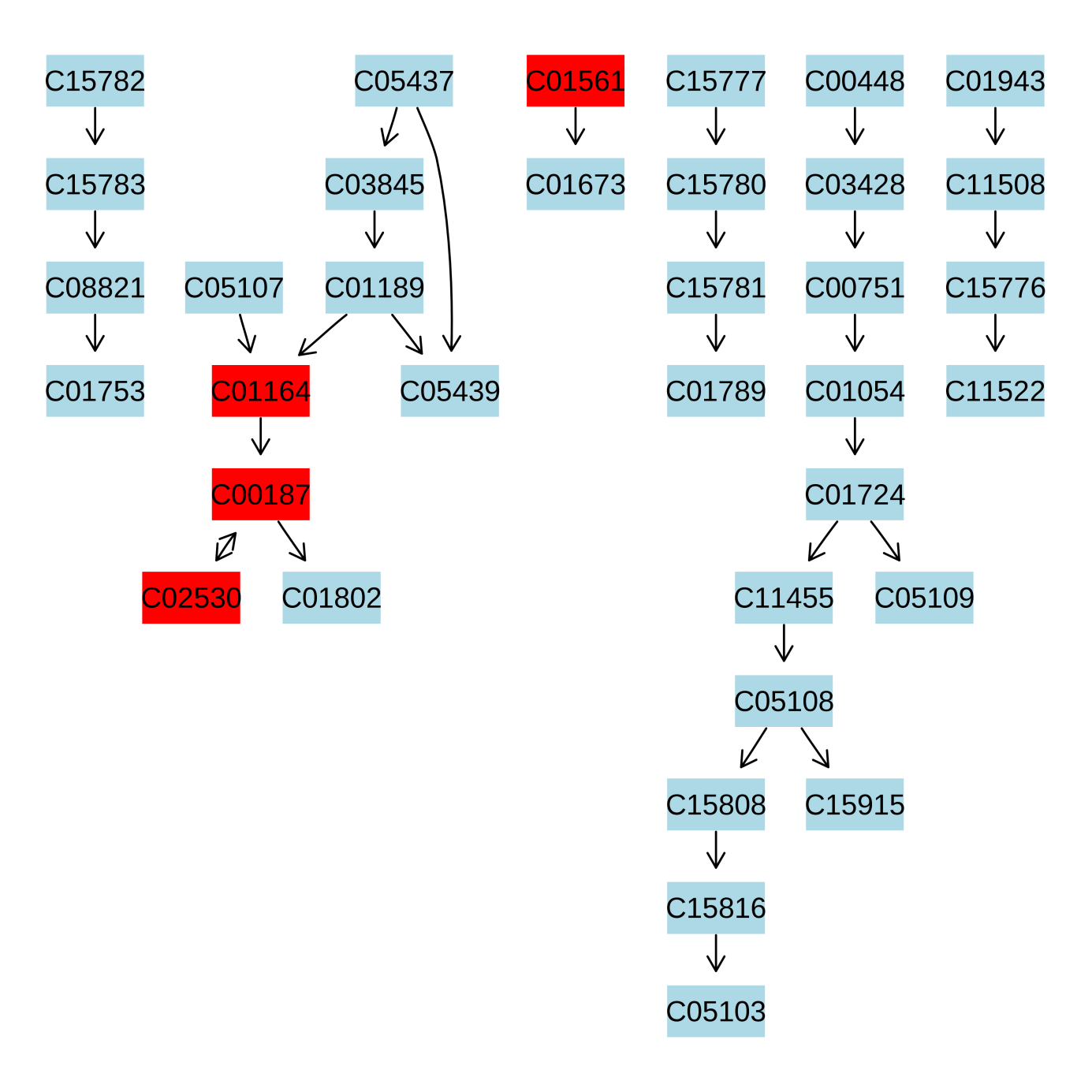


|  | **Hippocampus** | | | | | |  | **Plasma** | | | | | |
| --- | --- | --- | --- | --- | --- | --- | --- | --- | --- | --- | --- | --- | --- |
|  | **48h** | | **1wk** | | **12wk** | |  | **48h** | | **1wk** | | **12wk** | |
| **C01561: 25-Hydroxyvitamin D3** | 0 | 0 | 0 | 0 | 0 | 0 |  | -1 | 0 | -1 | 0 | 0 | 0 |
| **C01164: 7-Dehydrocholesterol** | 0 | 0 | -1 | 0 | -1 | 0 |  | 0 | 0 | 0 | 0 | 0 | 0 |
| **C00187: Cholesterol** | 0 | 0 | 0 | 1 | 0 | 0 |  | 0 | 0 | 0 | 0 | 0 | 0 |
| **C02530: Cholesterol ester** | 0 | 3 | 0 | 5 | -4 | 0 |  | 0 | 0 | 0 | 0 | 0 | 0 |
